# Second type of criticality in the brain uncovers rich multiple-neuron dynamics

**DOI:** 10.1073/pnas.1818972116

**Published:** 2019-06-12

**Authors:** David Dahmen, Sonja Grün, Markus Diesmann, Moritz Helias

**Affiliations:** ^a^Institute of Neuroscience and Medicine (INM-6), Jülich Research Centre, 52425 Jülich, Germany;; ^b^Institute for Advanced Simulation (IAS-6), Jülich Research Centre, 52425 Jülich, Germany;; ^c^JARA Institute Brain Structure-Function Relationships (INM-10), Jülich-Aachen Research Alliance, Jülich Research Centre, 52425 Jülich, Germany;; ^d^Theoretical Systems Neurobiology, RWTH Aachen University, 52056 Aachen, Germany;; ^e^Department of Psychiatry, Psychotherapy and Psychosomatics, School of Medicine, RWTH Aachen University, 52074 Aachen, Germany;; ^f^Department of Physics, Faculty 1, RWTH Aachen University, 52062 Aachen, Germany

**Keywords:** cortical dynamics, balanced state, criticality, correlated neural activity

## Abstract

Cortical networks that have been found to operate close to a critical point exhibit joint activations of large numbers of neurons. However, in motor cortex of the awake macaque monkey, we observe very different dynamics: massively parallel recordings of 155 single-neuron spiking activities show weak fluctuations on the population level. This a priori suggests that motor cortex operates in a noncritical regime, which in models, has been found to be suboptimal for computational performance. However, here, we show the opposite: The large dispersion of correlations across neurons is the signature of a second critical regime. This regime exhibits a rich dynamical repertoire hidden from macroscopic brain signals but essential for high performance in such concepts as reservoir computing. An analytical link between the eigenvalue spectrum of the dynamics, the heterogeneity of connectivity, and the dispersion of correlations allows us to assess the closeness to the critical point.

## Significance

Parallel recordings of motor cortex show weak pairwise correlations on average but a wide dispersion across cells. This observation runs counter to the prevailing notion that optimal information processing requires networks to operate at a critical point, entailing strong correlations. We here reconcile this apparent contradiction by showing that the observed structure of correlations is consistent with network models that operate close to a critical point of a different nature than previously considered: dynamics that is dominated by inhibition yet nearly unstable due to heterogeneous connectivity. Our findings provide a different perspective on criticality in neural systems: network topology and heterogeneity endow the brain with two complementary substrates for critical dynamics of largely different complexities.

The brain is a dynamical system with potentially different regimes of operation. Network models and experiments suggest optimal computational performance at critical points in parameter space, which mark the transition between two dynamical regimes (1). At such points, different systems exhibit the same universal behavior. For example, the transition from a liquid to a gas and the transition from a ferromagnet to a paramagnet follow the same quantitative description on large scales, despite their different structure at microscopic scales. The reason is that strong concerted fluctuations between all constituents lead to effective long-range interactions despite short-range couplings. The same phenomenon happens in neuronal networks.

A well-studied type of criticality occurs in neuronal networks with equal excitatory and inhibitory feedback, leading to neuronal avalanches (2), which are visible as large transients of population activity with a slowly decaying autocorrelation (3). Next to the original work in brain slices, signatures of avalanches have also been observed in motor cortex of awake macaques in mesoscopic measures of neuronal activity, such as the local field potential (4).

**Fig. 1.**
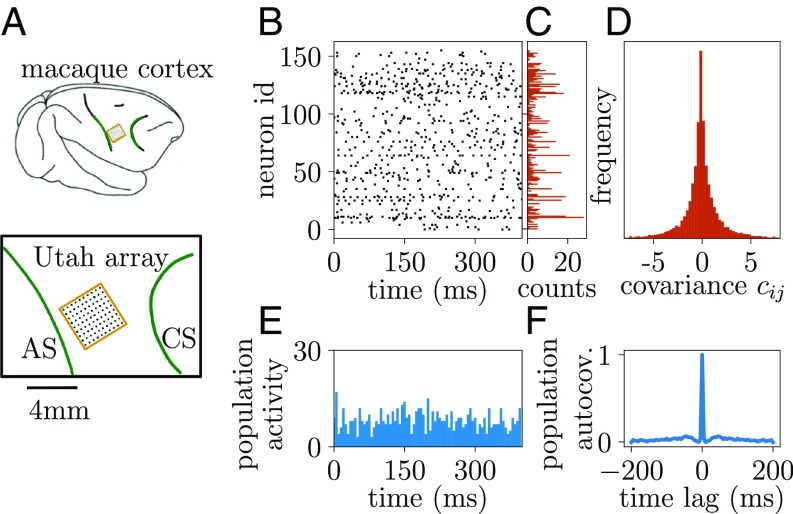
Spiking activity in macaque motor cortex. (*A*) A 10×10-electrode “Utah” array (Blackrock Microsystems; black dots) with 400-μm interelectrode distance covering an area of $4\times 4\;{mm}^{2}$ (yellow square) of macaque motor cortex between the arcuate (AS; left green curve) and the central sulcus (CS; right green curve) of the right hemisphere. (*B*) Single-trial raster plot of spiking activity of 155 neurons within T=400 ms after trial start of a reach-to-grasp task (5, 6). (*C*) Spike counts $n_{i}$ of activity within T=400 ms. (*D*) Distribution of covariances $c_{ij}=\frac{1}{T}\left(\left\langle n_{i}n_{j}\right\rangle -\left\langle n_{i}\right\rangle \left\langle n_{j}\right\rangle \right)$ between spike counts $n_{i}$ in 141 trials. (*E*) Time-resolved population activity binned in $t_{bin}=5\;ms$. (*F*) Autocovariance function of binned population activity.

Here, we consider parallel recordings of neuronal spiking activity in this cortical region. The data of the considered period neither show large transients of the summed spiking activity of all neurons termed population activity (Fig. 1 *B* and *E*), nor exhibit long correlation times (Fig. 1*F*). The data rather reveal weak and fast fluctuations of the population activity. This observation is in line with the network operating in the so-called balanced state (7): in this model architecture, the connectivity is endowed with an excess of inhibitory feedback so that a low level of activity arises that is dynamically stabilized. A consequence is that excitatory and inhibitory synaptic currents are anticorrelated (8), confirmed by network theory (9). Theory, moreover, predicts that correlations between pairs of neurons are weak on average (10). This is what we indeed observe in Fig. 1*D*: the distribution of correlations across all recorded neurons is tightly centered around zero.

**Fig. 2.**
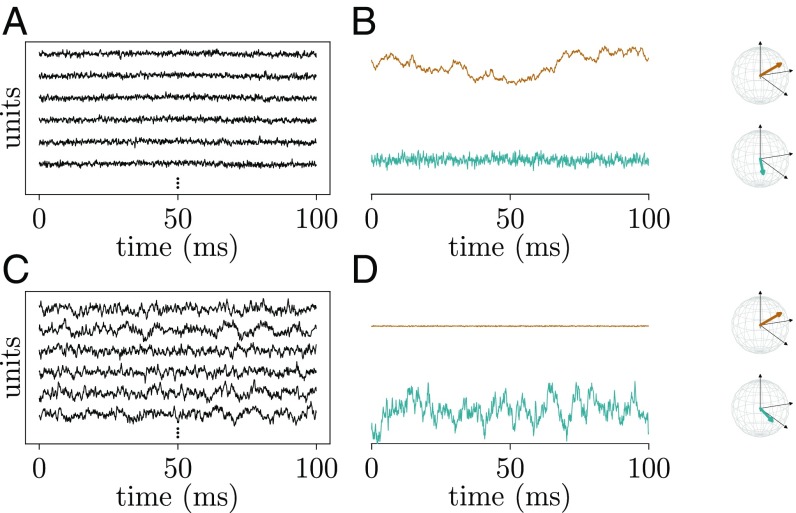
Example of multineuron coordinated activity. Networks of randomly interconnected linear units that show coordinated behavior either in the population activity as one particular state space projection (*A* and *B*) or in another projection (*C* and *D*). (*A* and *C*) Activity traces (black curves) of six example units of the N=1000-dimensional networks. (*B* and *D*) Projection of network activity of N units on different directions in the N-dimensional space illustrated by colored arrows in the coordinate systems (black) spanned by units 1–3. Orange, direction of population activity; cyan, direction of eigenvector with largest bulk eigenvalue of the connectivity matrix (Fig. 6).

Therefore, can we conclude that the network is operating far away from a critical point in a regime suboptimal for information processing? This conclusion would be too quick to make, because there is a second type of criticality devoid of avalanches, which has been investigated in computational studies (11): edge-of-chaos criticality. With increasing heterogeneity in network connections, the dynamics changes from regular to chaotic. At the transition to chaos, the network possesses a rich repertoire of coexisting and topologically complex multiple-neuron dynamics (12). These are coordinated changes of the activity of a large number of neurons, where each neuron increases or decreases its activity by a percentage that can differ from neuron to neuron. Therefore, the coordinated behavior might not be visible in the often-considered population activity (Fig. 2 *A* and *B*), which is only one specific projection of the high-dimensional neuronal activity. Instead, coordination between neurons can be observed in other directions that are not defined by the population identity of neurons but by the detailed microscopic structure of the network (Fig. 2 *C* and *D*). Identifying such directions in the data is obviously a daunting task, and therefore, we are in need of indirect indicators of such a state.

One candidate is stability of the dynamics. Here and throughout the remainder of the study, we call a system stable if the dynamics returns back to the same state after a perturbation. The simplest example is a fixed point: stable dynamics relaxes back to the fixed point after a small initial deflection. The onset of chaotic activity has been shown to coincide with the breakdown of stability of the fixed point of vanishing activity in deterministic dynamical systems (13). Up to a shift in the point of transition, the argument also holds in the presence of noise for the stability of the stochastic dynamics (14). Noise-driven networks show optimal computational performance in terms of memory capacity in the zone where the dynamics is unstable on short timescales but not yet chaotic (14). A direct measurement of stability is, however, hampered by noise and the sparse sampling of neurons in the local network. What is, therefore, needed is a reliable measure assessing how far the network operates away from the breakdown of stability.

What could constitute such a measure that can readily be observed in neuronal data? Clearly, it must be a quantity that is sensitive to the mediators of interactions between neurons, the synaptic connections, because these are the basis for collective behavior coordinated across many neurons. In this paper, we show that distributions of correlations in neuronal activity (Fig. 1*D*) provide a measure of stability (15–17). Applied to the massively parallel data from macaque motor cortex, our analysis shows that this brain region indeed operates close to a critical point that marks the breakdown of stability.

## Results

### Correlations as a Measure for Linear Stability.

It is well known that pairwise covariances in the activity of neurons, to a good approximation, follow a simple law c(W), which relates covariances $c_{ij}$ between all pairs of neurons to the effective connectivity matrix W of the network (15–17) (*Materials and Methods*). The latter is the product of the anatomical connectivity and the sensitivity of individual neurons. In consequence, $W_{ij}$ measures the change in firing probability of a postsynaptic neuron i due to a single spike of a presynaptic neuron j. For the covariance c that is integrated over the time lag, the relation c(W) is independent of the neuron model; it holds for networks of spiking model neurons as well as for binary model neurons and even for continuous rate dynamics (18). In addition, c(W) does not depend on synaptic delays and time constants.

The effective connectivity matrix W determines not only the covariances in the network but also, the stability of the network dynamics. The latter becomes unstable if one eigenvalue of W has a real part Re(λ)≥1. Eigenvalues are, in principle, determined by all connections in the network. However, their determination from experimentally observed covariances is also hindered by severe subsampling; even with massively parallel recording techniques, at most hundreds of neurons can be measured simultaneously from the same local network. An inversion of the relation c(W) is, therefore, not feasible. Thus, we need to find a signature in the correlations that is informative about the eigenvalues, that is insensitive to the details of the network, and that can be estimated from a few hundred neurons at most.

A striking feature of the correlations is their low mean across the population (Fig. 1*D*), which has already been investigated using population-averaging techniques (9). This low mean has been shown to relate to a single highly stable eigenvalue λ with Re(λ)<0 (10); the network is said to be inhibition dominated. A priori, the mean provides no information on the other N−1 eigenvalues in a network of N neurons. Thus, it is necessary to study the distribution of covariances beyond its mean. Such analysis is, however, not possible with either existing population averaging or mean field techniques.

To proceed, we turn to ideas from disordered systems. These are systems with parameters that are drawn randomly: in our case, the connectivity of the network. If sufficiently large, these systems often show the remarkable feature of being self-averaging: that is, their macroscopic state does not depend on the precise value of each of their random parameters but rather, only on their statistics. Spin glasses are the most prominent example (19). Transferring this idea to the problem at hand, we need to give up on the pursuit of any neuron in its individuality, but we rather need to ask how the correlations are distributed across the measured set of neurons. Technically, we implement this idea by extending dynamical mean field techniques (20) combined with methods from disordered systems (19). Going beyond the commonly applied mean field theory for auxiliary fields (14, 21), in *SI Appendix*, we show that the leading order fluctuation corrections can be related to the distribution of covariances within a single-network realization. The resulting closed form expressions provide a direct relation between the width of the distribution of cross-covariances and the stability of the dynamics as measured by the largest eigenvalue (spectral radius) of the effective connectivity matrix (*Materials and Methods*). The theory reproduces that mean covariances are low if the network is inhibition dominated (9, 10). Furthermore, it predicts that, for large spectral radii, the width of the distribution of covariances is much larger than the mean as experimentally observed in our data (Fig. 1*D*).

**Fig. 3.**
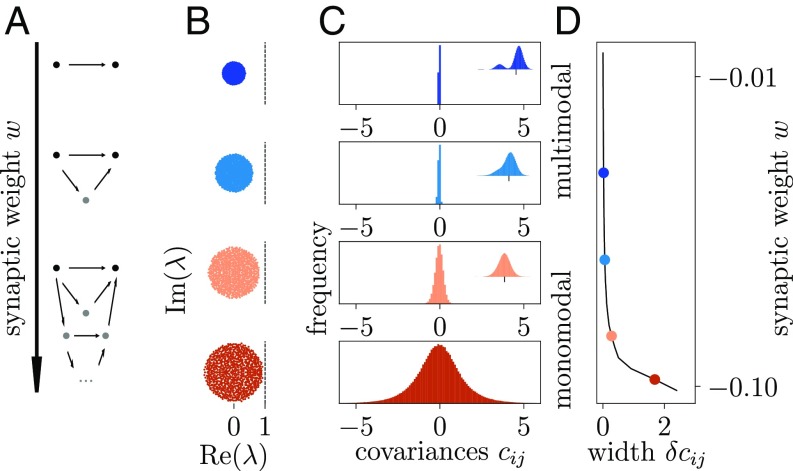
Dispersion of correlations measures stability of network dynamics. Connection strength w in a sparse random network increases from top to bottom (vertical axis). (*A*) Connections (arrows) between a pair of observed neurons (black dots); indirect connections contribute to correlations via intermediate neurons (gray dots). (*B*) Bulk eigenvalues λ (colored dots) of the connectivity matrix W in the complex plane [critical line at Re(λ)=1]. (*C*) Distributions of covariances. Enlargements are shown in *Insets*. (*D*) SD $\delta c_{ij}$ of distribution of covariances (black curve; colored symbols correspond to distributions shown in *C*).

The theoretical predictions are intuitive as highlighted by the dependence of the distribution of covariances on the strength of connections in a sparse inhibition-dominated network (Fig. 3). With increasing connection strength, activity successively propagates over multiple synapses, leading to indirectly mediated interactions via a growing number of parallel paths (Fig. 3*A*) (16, 17). For weak synapses, only direct connections matter, and covariances are mainly determined by the presence or absence of a connection between two neurons. However, as the synaptic amplitude grows, the effect of one neuron on another is also exerted indirectly via one or multiple intermediate neurons.

Varying the strength of the nonzero connections is tantamount to changing the variance of the entries in the connectivity matrix. The radius of the cloud of its eigenvalues depends directly on this variance, which quantifies the heterogeneity across connections (22). The larger the eigenvalues of the connectivity matrix, the more important are contributions with a high number n of intermediate neurons; such a contribution is proportional to $W^{n}$. As the radius in Fig. 3*B* approaches unity, implying an eigenvalue with Re(λ)=1 that causes unstable dynamics, indirect paths of any length n become as important as direct ones. As a consequence, distributions of covariances become monomodal and broader but stay centered approximately at zero (Fig. 3 *C* and *D*). At the same time, this point is precisely where the dynamics loses stability. These results hence expose a hallmark of dynamically balanced networks that operate close to instability: widely distributed covariances with a small mean as we observe in the motor cortex (Fig. 1*D*).

### Inference of Operational Regime.

**Fig. 4.**
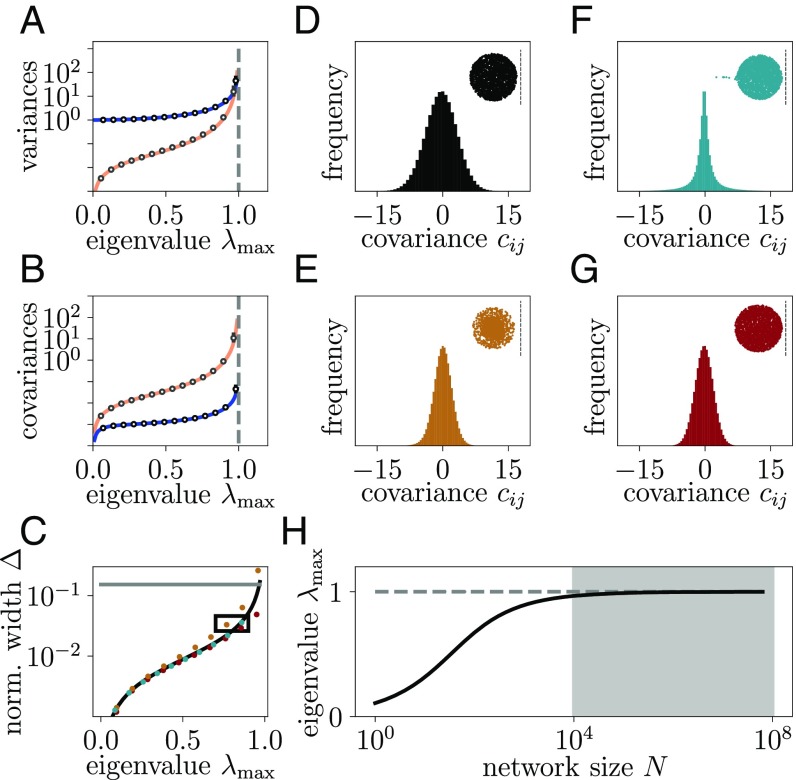
Large width of covariances reveals dynamics of motor cortex close to instability. (*A* and *B*) Theoretical prediction for the mean (Eq. **4**; blue curve) and SD (Eq. **5**; orange curve) of variances (*A*) and covariances (*B*) for different maximum eigenvalues $\lambda_{\max}$ compared with numerical results (Eq. **3**; markers) for homogeneous inhibitory networks. Ensemble averages for 100 network realizations (circles) and average upward/downward deviation of the non-Gaussian statistics (bars). Bars are below marker size, except for rightmost data points. (*C*) Theoretical prediction for the normalized width Δ (black curve) of covariances compared with numerical results (Eq. **3**; markers) for excitatory–inhibitory networks (orange), inhibitory networks with distance-dependent connectivity (cyan), and direct simulations of inhibitory networks of spiking leaky integrate-and-fire neurons (red). Distributions corresponding to data points within the black rectangle are shown in *D–G*. The gray horizontal line indicates value Δ=0.15 corresponding to moments of the covariance distribution measured in macaque motor cortex (Fig. 1). (*D–G*) Distribution of covariances (histogram) and bulk eigenvalues (dots) for a homogeneous inhibitory network model (*D*), for a network of excitatory and inhibitory neurons (*E*), for a network with distance-dependent connection probability (*F*), and for a homogeneous inhibitory network of spiking leaky integrate-and-fire neurons (*G*). Dashed lines indicate the critical value $Re\left(\lambda_{\max}\right)=1$. (*H*) Predicted maximum eigenvalue $\lambda_{\max}$ (Eq. **1**) of the effective connectivity of macaque motor cortex as a function of the number of neurons N for given Δ=0.15. The shaded area marks the range of biologically plausible effective network sizes corresponding to the spatial scale of the recordings.

How can we use these results to infer how close to instability the cortical network operates? Our theory, in principle, determines the largest eigenvalue from measured covariances. However, the theoretical derivations assume a homogeneous random network and linear model neurons. It is thus unclear how robust the result is with regard to more realistic characterizations of cortical connectivity and dynamics. We, therefore, investigate numerically more complex network topologies, such as excitatory–inhibitory networks fulfilling Dale’s law (Fig. 4*E*) and networks with distance-dependent connection probabilities (Fig. 4*F*). In *SI Appendix*, we further compare the theoretical predictions with networks with other sources of heterogeneity, such as a log-normal distribution of synaptic weights, a log-normal firing rate distribution, correlated external inputs, and correlated connections. We find that the higher-order cumulants of the distribution of covariances can be sensitive to these more complex network features—for example, networks with spatially dependent connectivity most closely match the shape of the experimentally observed covariance distribution with a pronounced peak close to zero (Fig. 4*F*). However, the normalized width of the distribution of covariances $\Delta =\delta c_{ij}/c_{ii}$ turns out to be robust against these complex features (Fig. 4*C*). As predicted by the theory, the most unstable bulk eigenvalues determine the width of the distribution. They result from the heterogeneity of network connectivity that mainly arises from the sparsity of connections, common to all considered network models (Fig. 4 *D*–*G*) and a well-known feature of local cortical connectivity. By comparing the theoretical results with direct simulations of spiking networks (23), we further confirm the applicability of the theory beyond the linear response approximation (Fig. 4*G*). The use of spiking networks further illustrates that a range of Δ covering two orders of magnitude can be achieved through variation of synaptic strength by roughly a factor 10 within the biologically plausible (24) and commonly chosen (25) regime [0.1,1.1] mV (Fig. 4*C*). These networks have eigenvalues $\lambda_{\max}$ between 0.1 and ≈1; the range, therefore, encompasses networks that are far away from criticality and networks that are close to criticality. As a consequence of this general applicability of the theory, the prediction for the normalized width Δ can be used to infer the operational regime of the cortical network (Fig. 1).

The distance to instability is determined by $\lambda_{\max}$, which to leading order in the network size N, is given as (*Materials and Methods*)

$$\lambda_{\max}=\sqrt{1-\sqrt{\frac{1}{1+N\Delta^{2}}}}.$$ [1]

With the biologically plausible estimate of at least $N=10^{4}$ neurons below the recording Utah array and together with the measured normalized width Δ=0.15 (from Fig. 1, with bias correction due to the finite amount of measured data, and *SI Appendix*), this leads to a substantial quantity $N\Delta^{2}$ such that Eq. **1** predicts the largest eigenvalue $\lambda_{\max}≲1$ close to the critical value of one (Fig. 4*H*, gray area). From this eigenvalue and from the low mean covariance in the data (Fig. 1*D*), we conclude that the biological network operates in a dynamically balanced critical regime with nearly unstable dynamics.

### Dynamically Balanced Critical Regime Vs. Critical Population Dynamics.

**Fig. 5.**
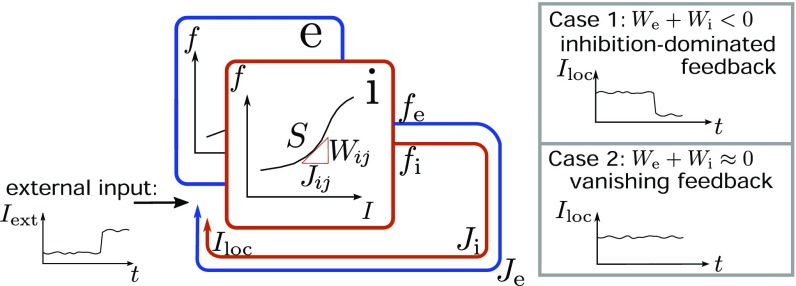
Classification of networks based on feedback of population activity. A perturbation of excitatory (blue) and inhibitory (red) populations of neurons by an external input $\delta I_{ext}$ (black time course) causes changes in firing rates $\delta f=S\cdot \delta I_{ext}$ and recurrent inputs $\delta I_{loc}=J_{e}\delta f_{e}+J_{i}\delta f_{i}=\left(W_{e}+W_{i}\right)\;\delta I_{ext}$ via excitatory ($J_{e}$; blue arrow) and inhibitory ($J_{i}$; red arrow) local connections. The recurrent input counteracts the change in the external input (Case 1; inhibition-dominated feedback; upper gray box), remains unaffected (Case 2; vanishing feedback; lower gray box), or amplifies the external perturbation (locally unstable).

**Fig. 6.**
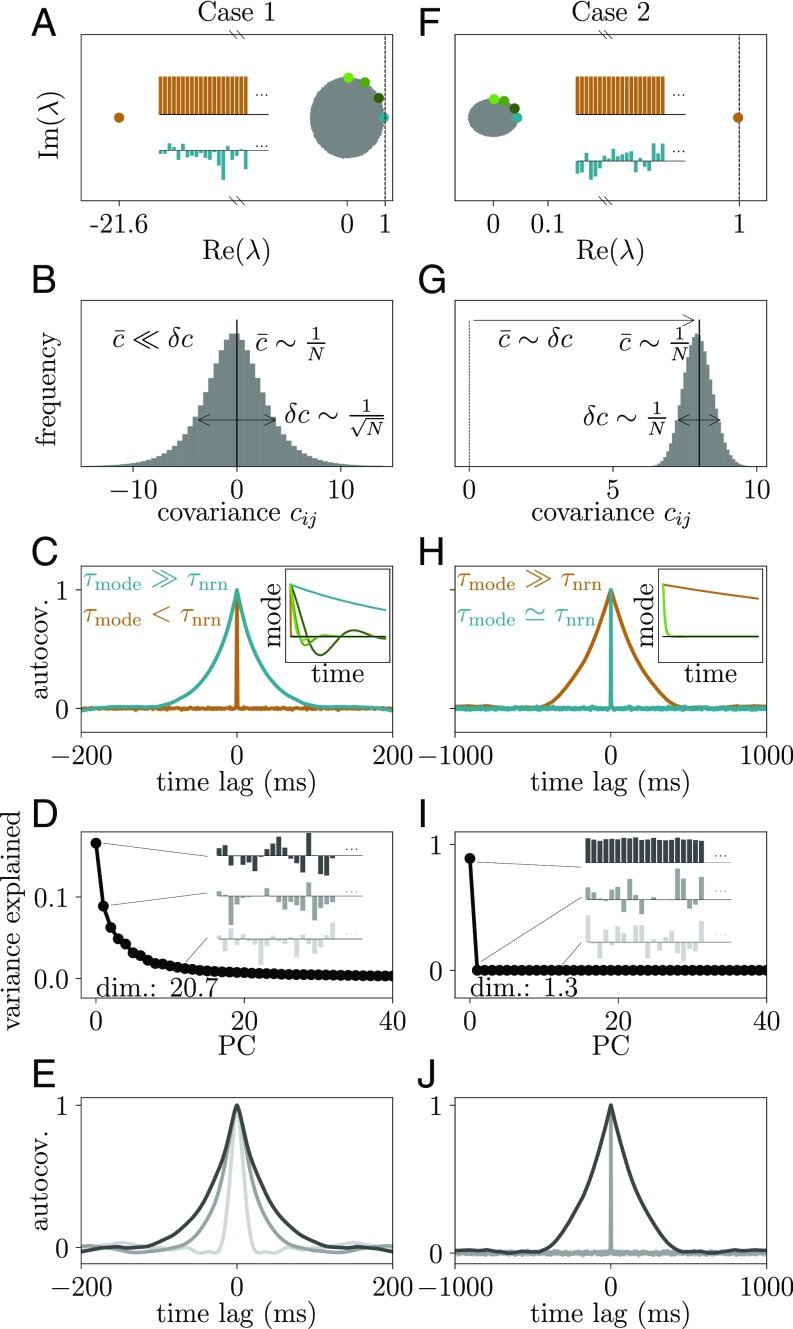
Two types of criticality. Activity statistics on the level of individual neurons in networks with inhibition-dominated or vanishing feedback (Cases 1 and 2 of Fig. 5, respectively) in the critical regime. (*A–E*) Case 1: dynamically balanced network with stable population activity but virtually unstable linearized dynamics hidden in specific linear combinations of neuron activities. (*A*) Spectrum of eigenvalues with negative outlier (orange dot) and nearly critical bulk eigenvalue (cyan dot) and corresponding eigenvector (cyan bars) generated by heterogeneity in connections across neuron pairs. (*B*) Distribution of covariances with almost vanishing mean and large SD. (*C*) Autocovariance functions of the population activity (orange curve; compare with the orange dot in *A*) and the activity projected onto the eigenvector corresponding to the largest real bulk eigenvalue (cyan curve). (*Inset*) Time course of network modes corresponding to colored eigenvalues in *A*. (*D*) Normalized eigenvalues of PCA (black dots) and loadings of eigenvectors for components 1,2,13 (bar plots). (*E*) Autocovariance functions of activity projected onto PCs 1,2,13 (same color code as in *D*). (*F–J*) Case 2: network with almost vanishing excitatory and inhibitory feedback and virtually unstable linearized population dynamics. (*F*) Spectrum of eigenvalues with positive outlier (orange dot) and corresponding eigenvector (orange bars; population activity) generated by average connectivity structure. (*G*) Distribution of covariances with positive mean and small SD. (*H*) Autocovariance functions of the population activity (orange curve) and the activity projected onto the eigenvector corresponding to the largest bulk eigenvalue (cyan curve; compare with the cyan dot in *F*). (*Inset*) Time course of network modes corresponding to colored eigenvalues in *F*. (*I*) Normalized eigenvalues of PCA (black dots) and loadings of eigenvectors for components 1,2,13 (bar plots). (*J*) Autocovariance functions of activity projected onto PCs 1,2,13 (same color code as in *I*).

What does the dynamically balanced (Fig. 5, case 1) critical regime imply for the activity of the network? In general, a value $\lambda_{\max}≲1$ implies that a large number of eigenvalues of the effective connectivity matrix must be close to the critical line where stability breaks down (Fig. 6*A*). Each eigenvalue is associated with a unique direction in the N-dimensional space of neurons (compare with Fig. 2), the eigenvector of the effective connectivity matrix. The dynamics along this direction (mode; i.e., the projection of the activity onto this eigenvector) involves all N neurons, each in proportion to their loading in the eigenvector. Fig. 6*A* shows an example. Some neurons participate in this collective dynamics with positive sign (positive loading), and some participate with negative sign (negative loading). The signature of such a mode is hence that all neurons change their activity in a concerted manner: some by increasing and others by decreasing their activity (compare with Fig. 2*C*). Those modes that have eigenvalues on the right edge of the circle, close to the critical line, in addition have slow dynamics. Changes of activity of such modes evolve on considerably longer timescales $\tau_{mode}=\tau_{nrn}/\left(1-\lambda \right)$ compared with the timescale $\tau_{nrn}$ on which a single neuron typically changes its activity. This is visible in a slowly decaying autocorrelation function (Fig. 6*C*). The typically considered population activity is only one particular mode that weights all neurons equally. The almost vanishing mean of the covariances (Figs. 1*D* and 6*B*) and the weakly fluctuating and quickly decaying population activity (Figs. 1*F* and 6*C*) show that the corresponding population eigenvalue (Fig. 6*A*, orange dot) is negative (i.e., the feedback is inhibition dominated) (9, 10, 26) (Fig. 5, Case 1). Having a multitude of eigenvalues with arbitrarily small distance to the critical line implies rich multiple-neuron dynamics with largely different time courses (Fig. 6*C*). These time courses define the response repertoire of the network that would be visible when stimulating in the direction of the eigenvectors of the effective connectivity.

How is the richness of the response repertoire related to the ongoing dynamics? For the experimental data, the effective connectivity and its eigenvectors are not known. Fig. 6 *A* and *F* shows that all eigenvectors apart from the population mode have complex structure, which derives from the heterogeneous effective connectivity matrix. Therefore, the above response repertoire cannot be easily measured. Still, one can indirectly infer properties of the response repertoire based on the ongoing dynamics and vice versa. For example, if there were no slow response modes, there could not be slow dynamics in any direction of the *N*-dimensional space of neurons. In contrast, if there is a multitude of nearly unstable modes, dimensionality reduction techniques should be able to find directions in state space with slow dynamics. Indeed, from linear systems analysis follows directly that not only the timescale of fluctuations increases close to the critical line but also the magnitude. Therefore, a principal component analysis (PCA) that searches for directions in state space with maximal variance would predominantly detect the directions with slow dynamics. The eigenvalues of the matrix of time-lag integrated covariances in the dynamically balanced critical regime have a continuum of distances to the critical line and therefore, imply a continuous spectrum of principal components (PCs) (Fig. 6*D*) and a gradual decrease of timescales with increasing PC (Fig. 6*E*). As a result, the ongoing dynamics of the network is multidimensional. However, since the variance of the mode dynamics scales as $1/{\left(1-\lambda \right)}^{2}$, the rightmost eigenvalues dominate such that the dimensionality, measured by the participation ratio (27, 28), is low compared with the number of neurons in the network.

**Fig. 7.**
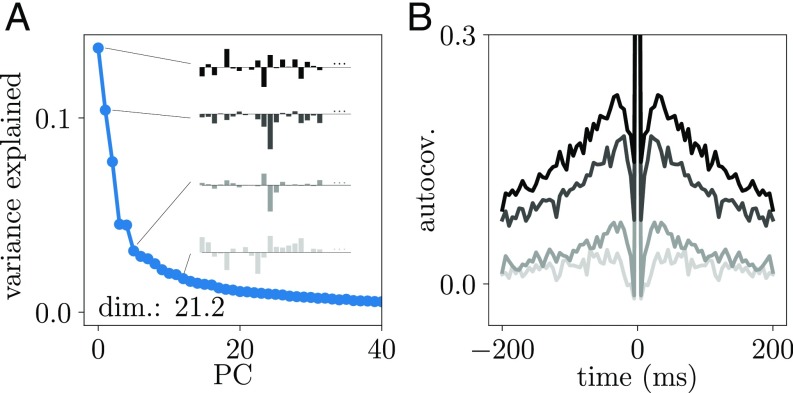
PCA of motor cortex data. (*A*) Fraction of variance explained as a function of the PC index. Dimensionality dim=21.2 measured by the participation ratio (27). Bar plots show loadings of selected PCs (indicated by black lines). (*B*) Autocovariance function of activity projected onto selected PCs (same color code as in *A*).

The predictions of our theory for timescales and dimensionality based on the distribution of covariances can be readily tested in the experimental data at hand. Applying a PCA on the macaque motor cortex spike counts indeed reveals a low dimensionality of the dynamics and a continuous spectrum of PCs (Fig. 7*A*). Projecting the spiking activity onto the PCs shown in Fig. 7*A* yields a temporal sequence, of which we analyze the autocovariance function in Fig. 7*B*. All autocovariances exhibit a central peak arising from the spiking nature of the signals. The timescale of the smooth part of the autocorrelation function is large for the first PC and gradually decreases with increasing PC (Fig. 7*B*) as predicted by the theory of the linear rate model. This shows that temporal features of the dynamics on behavioral timescales are imprinted in the effective connectivity of the network.

Having discussed the features of this dynamically balanced critical state, one may wonder how it is related to the known state of avalanche criticality. Avalanche criticality (29–31) appears in networks with nearly equal excitatory and inhibitory feedback (μ≈1/N) (Fig. 5, Case 2). The strongly fluctuating population activity observed in such networks causes positive covariances (Fig. 6*G*) and a slowly decaying autocorrelation function of the population activity (Fig. 6*H*). These dynamics are determined by the single nearly unstable eigenvalue of the population activity that results from the average connectivity structure of the network (32) (Fig. 6*F*, orange dot). Each of the remaining N−1 modes has a low amplitude and exponentially decaying, fast dynamics (Fig. 6 *H* and *J*). Thus, the network in such a critical state is effectively one-dimensional (33) as reflected by a single outlier PC (Fig. 6*I*). Due to the distinguished role of the population eigenvalue, the mode with slowest dynamics coincides with the first PC, as all other modes are almost negligible. This makes it possible to detect such criticality in population-averaged or coarse-grained measures of neural activity. A summary of qualitative features of the two critical states is given in Table 1.

**Table 1.** Qualitative features of the two critical states

Feature	Case 1	Case 2
Population activity		
Fluctuations	Fast and weak	Slow and strong
Spike count covariance		
Mean	Low	High
Width	Large	Small
Stimulation		
Whole population	Fast and small response	Slow and strong response
Specific direction (mode)	Various timescales and amplitudes	Stereotypic timescale and amplitude
PCs		
Eigenvalues	Gradual decline	Single dominant eigenvalue
Dominant eigenvectors	Heterogenous loadings	Homogeneous loadings
Dimensionality	1<dim≪N	1≲dim

Case 1 (dynamically balanced criticality) and Case 2 (avalanche criticality) correspond to the definitions in Fig. 6.

## Discussion

In this study, we reconcile the contradiction between experimentally observed weak pairwise correlations and the presence of a critical state, which is assumed to be necessary for efficient information processing. We show that a linear model explains the on average weak pairwise correlations and the wide dispersion across cells that we observe in massively parallel spike recordings of macaque motor cortex. Comparing experimental data with theoretical predictions from network models, in general, faces a subsampling problem. Only a tiny fraction neurons in a local circuit is recorded at a time. We here overcome this problem by deriving a theory that relates the statistics of correlations of the spiking activities to the statistics of connections. This relation exposes a simple and robust correspondence between the width of the covariance distribution and the stability of the network dynamics. The distribution of correlations in the macaque data can only be explained by a network dynamics with a multitude of nearly unstable modes and a single-population mode that is stabilized by inhibition. Such a dynamically balanced critical state entails that motor cortex has a rich dynamical repertoire at its command, which is, however, invisible in the commonly considered population-averaged activity. This hidden response repertoire explains the shape of PC spectra, the low dimensionality in the data, and the timescale of dynamics in PC space.

### Unified View of Criticality in High-Dimensional State Space.

The found dynamically balanced criticality is contrasted with the established notion of avalanche criticality in neuronal networks by Table 1. However, the two types of critical states are not mutually exclusive, as they are governed by different mechanisms. The overall level of excitation and inhibition in the network controls the population activity and can be adjusted to obtain avalanche criticality (2). Heterogeneity in the network shapes the spectral radius of the effective connectivity, which close to unity, causes criticality of the here found second kind that is visible in specific directions in the high-dimensional state space. Therefore, critical dynamics of both kinds can coexist in different brain regions or even in the same local network. Changing the overall level of activity in networks changes the sensitivity of neurons to inputs and therefore, the effective connectivity (34, 35). By this mechanism, networks may be dynamically moved into different dynamical regimes to adapt brain function to momentary demands.

Many studies have presented experimental evidence for avalanche criticality in the brain (1, 36). Likewise, there is a vast amount of literature on the dynamically balanced state of cortical networks (8, 9, 26, 37). Proponents of either dynamical regime are split into two disjoint camps, as the notion of criticality seemed to contradict the asynchronous irregular activity in the balanced state. This study, however, unifies the two concepts and shows evidence of criticality in the latter state. We show that the nature of both types of criticality can be explained with eigenvalues at the edge of stability, which cause strong transients of activity. Although the mechanism is the same, the different directions of the critical modes in state space and the different number of modes that have critical dynamics lead to qualitatively distinct dynamical states.

The typically considered population activity represents one particular projection of the high-dimensional neuronal state. Avalanche criticality would be detected in long time constants of the autocorrelation function of this projection (3). On the level of individual neurons, it would lead to pronounced positive average covariances across pairs of neurons (Fig. 6*G*) (33). Neither feature is observed in the motor cortex data of the awake macaque monkey considered here. Instead, we find signatures of the dynamically balanced state: short time constants of the population autocorrelation function and average pairwise covariances close to zero. Avalanche criticality cannot occur in this state, as fluctuations of the population activity are small, likely suppressed by negative feedback (10). As a result, networks show fast tracking between excitatory and inhibitory synaptic currents (8, 9) and between the activities of different populations (38).

Finally, the overall level of excitation and inhibition and the heterogeneity in the network are not the only mechanisms that can lead to eigenvalues close to the critical line. Fine tuning of connectivity as well as sophisticated plasticity mechanisms can also move single eigenvalues or even large numbers of eigenvalues close to instability, leading to critical dynamics in certain directions in state space as shown by the seminal work of Hennequin et al. (39).

### Relation to Previous Theoretical Studies.

The recent discussions on criticality in neuronal networks have in common that specific models are studied with two main recurring themes. First, the idea of a branching parameter, specifically the average number of downstream descendants produced by the current state of activity, relates the emergence of a second-order phase transition to a one-dimensional model (40). Second, experimentally measured activity is compared with statistical models in thermodynamic equilibrium, such as pairwise maximum-entropy models. A common criticism of the first perspective is its coverage of only a small subset of all possible critical states; a wealth of physical systems shows various phase transitions that differ from a branching process (41). This view is in particular incompatible with the strong evidence for neuronal networks operating in the balanced state, in which inhibitory feedback dominates; the branching parameter would be effectively negative in these systems and thus, criticality impossible. A common critique of the second approach is that it compares the dynamical activity of a neuronal network that certainly operates out of thermodynamic equilibrium with an equilibrium ensemble. Phase transitions in nonequilibrium dynamical stochastic systems are generally quite different from those observed in thermodynamic equilibrium. Understanding such dynamical phase transitions, as pioneered by ref. 42, is still a field of active research in statistical physics (41). The discrepancy between the dynamics of a network and the currently used static mathematical description has been discussed in the literature. Mora and Bialek (43) state that the currently used mathematical language of equilibrium statistical mechanics is insufficient to describe criticality in dynamical systems and that efforts need to be undertaken to address the relations between statistical and dynamical criticality more clearly.

This work is a humble step in this direction. Making use of linear response theory that precisely captures correlations in asynchronous irregular neural network states, we consider the high-dimensional nonequilibrium dynamics of neural networks for which a well-studied relation between covariances and effective connections holds. This relation allows us to infer critical dynamics also in state space projections different from the population activity considered in avalanche criticality analyses and to make predictions for the dynamical repertoire of networks in high-dimensional state space. The correspondence between covariances and effective connections has been investigated before to relate network structure and dynamics (16, 17). However, a direct comparison with experimental data has so far been impossible due to the subsampling problem. Algorithms for network inference with hidden units have been developed (44, 45) but still cannot be applied reliably with the capabilities of current recording technology.

### Generality of the Findings.

Although the theoretical derivation, for simplicity, is based on homogeneous random networks, the relation between the stability and the normalized width of the covariance distribution holds more generally for asynchronous states that arise from the dominance of inhibition. This is confirmed numerically for excitatory–inhibitory networks fulfilling Dale’s law and for networks with distance-dependent connection probability. In addition, we validate our results in the presence of other sources of variability, such as correlated external input and distributions in firing rates, as well as log-normal or correlated synaptic weights. Furthermore, the validity of the linear response approximation for the calculation of the normalized width Δ in the full range of spectral radii is ensured by comparison with direct simulations of spiking leaky integrate-and-fire networks. The linearization works well as long as individual synapses have only a small impact on the firing of a cell. This assumption is, for example, violated in the state discussed in ref. 46. Its signatures, however, are not found in the motor cortex data.

The observation of high-dimensional criticality is not necessarily specific to macaque motor cortex. Experimental evidence for the operation of cortical networks in the dynamically balanced state is overwhelming (8, 38, 47). In addition to the low average covariances in this state, other cortical areas, such as visual cortex (37), also show a covariance dispersion comparable with our data (*SI Appendix*). The same analysis can be applied to different cortical areas and experimental conditions to determine their operational point $\lambda_{\max}$.

### Computational Implications of the Dynamically Balanced Critical Regime.

The operation in the dynamically balanced critical regime ($\lambda_{\max}≲1$) has several implications for learning and information processing. Neurons belonging to a critical mode show pairwise covariances that strongly exceed the average (Fig. 6*B*). Such covariances, in turn, are known to influence the synaptic connectivity by spike timing-dependent synaptic plasticity (48, 49). The wide distribution implies that some neurons exhibit strongly correlated activity—positive or negative; thus, synapses connecting these neurons will experience strong changes of synaptic amplitudes: the neuronal dynamics and the synaptic dynamics are hence tightly coupled in such states.

Another advantage of the dynamically balanced critical regime is adaptivity. Weak external inputs to the network are sufficient to shift a large number of eigenvalues across the edge of stability (12) and thereby, drastically change the recurrent network dynamics. Critical modes have a multitude of characteristic shapes and lifetimes (Fig. 6*C*, *Inset*) (39); they arise despite the stereotypical and fast dynamics of individual neurons as a result of the heterogeneity of the network. The rich repertoire enables the parallel integration and maintenance of signals over prolonged timescales. Such networks provide a wealth of transformations on the input and, therefore, support the concept of reservoir computing (50, 51).

A wide dispersion of the spectrum of the connectivity matrix may also have important consequences for the controllability of the network dynamics by external signals. The Gramian of a linear system, equivalent to the equal time covariance matrix of an Ornstein–Uhlenbeck process (52), determines the strength of a signal required to move the network dynamics into a desired target state (53). This is a potentially important property for computation: for example, for classification of temporal sequences. The spectral properties of the Gramian are tightly related to the eigenvalues of the effective connectivity (52). Future work should, therefore, investigate the implications for controllability of cortical dynamics with close to unstable eigenvalues as we have found.

### Identification of Relevant State Space Projections.

In the dynamically balanced state, the population activity is thus not informative about the coordination between neuron activities beyond the overall negative feedback. An obvious aim is, therefore, to identify the relevant projections in state space (i.e., those projections with large temporal variability on various timescales). Finding the modes directly from the data seems impossible; it requires inference of the full effective connectivity matrix from the subsampled data. Although challenging, an intriguing route to follow instead is to apply perturbations to the network state in different state space directions to actively probe the response repertoire of the network (54) (Fig. 6). The realization in an experimental setup is challenged by the selective stimulation of individual modes. Compound stimulations typically result in superimposed responses of many modes and would thus lead to an overall similar time course for different stimulation directions. Selective stimulation involves different levels of excitation and inhibition applied to each neuron, which in parts, becomes more and more feasible using optophysiology (55). To disentangle superimposed responses of several modes, one would need to devise blind source separation techniques (56) that specifically take into account the stereotyped damped oscillatory time courses of the response modes.

A simpler and more standard technique of dimensionality reduction is the PCA. It finds relevant projections in state space that contribute largest to the temporal variability. Although it has been used frequently to infer low-dimensional trajectories in state space that correlate with behavior (57) and to assess the dimensionality of the dynamics (27, 28), it is not directly showing the independent dynamical responses that a network can perform: the typically asymmetric effective connectivity and the symmetric covariance matrix have different eigenvectors, and therefore, the independent network responses evolve along directions that differ from the PCs. However, PCs are linear combinations of modes after all. Therefore, they can only show dynamics that is contained in the response repertoire. In particular, if the latter does not contain slowly decaying responses, then slow ongoing fluctuations cannot be present in the PCs.

PCA applied to spike counts from macaque motor cortex recorded during the pretrial period of the reach-to-grasp task indeed shows that a multitude of PCs contributes with comparable weight. The shape of the PCA spectrum resembles the one of a dynamically balanced critical model network. As predicted by the theory, the timescale of each PCA component covaries with its importance: a component that provides a stronger contribution to the variance also has a slower timescale. The loadings of each mode, the coefficients with which each neuron contributes, are indeed highly complex, again consistent with the state of criticality arising from the heterogeneous connectivity rather than from a single outlier of the spectrum as in the case of avalanche criticality. The here used PCA is based on the same data as the distribution of covariances, namely the matrix of spike count covariances. The relation between a wide dispersion of the latter and a low dimensionality of the dynamics as well as predictions for the timescales and for the directions of leading PCs follow from our theoretical arguments. Finding these theoretically predicted features of the PCA in the data, therefore, substantiates our inference of the dynamically balanced critical regime.

A possible alternative approach to the here chosen time lag integrated covariances is measuring the matrix of equal time covariances. For an Ornstein–Uhlenbeck process, this matrix is identical to the Gramian matrix. Relations between the spectrum of the connectivity and the spectrum of the Gramian are known (52) so that one could directly relate the PCA spectrum of the equal time covariance matrix to the spectrum of the connectivity. There are, however, two major challenges to this approach. First, while the time lag integrated covariance is independent of many details of the network model (18) and thus, allows us to quantitatively compare theoretical results with experimental data and realistic spiking network models, the equal time covariance matrix depends on many such model details. A direct comparison is, therefore, more difficult. Second, the known relations between the spectra of connectivity and the Gramian hold for the entire matrices. In an experiment, however, we only observe a severely subsampled fraction of the entire covariance matrix. A comparison, therefore, requires the additional step of extrapolating the spectral properties of the entire covariance matrix from its subsampled estimate (28).

The dynamically balanced critical state leads to an entire region of eigenvalues of the effective connectivity that are close to unstable; a circular segment of eigenvalues has a real part close to unity. Every pair of modes with nonzero imaginary parts corresponds to a damped oscillatory temporal dynamics. Small changes of the effective connectivity, by synaptic plasticity or by external input, are then sufficient to move such a pair even closer to instability, making it the dominant mode. The resulting dynamics would show an exponentially damped oscillation. Such dynamics is indeed observed in PCs of motor cortex activity of behaving monkeys (57). It leads to a rotational behavior in PC space that is, furthermore, observed across various different tasks. This is a first indication for the functional relevance of the here-described state. The dynamics derived from the ongoing critical activity also shows up in task-related states. This suggests that motor cortex has a dynamic repertoire at its command that can be functionally used and differently composed in various tasks. Therefore, future studies should not only consider low-dimensional projections of task-related firing rate changes but also, scrutinize the fluctuations around stationary ongoing states. Similarities between these two conditions would strengthen the here-stated hypothesis that the low-dimensional dynamics on behaviorally relevant timescales is composed from the intrinsic vocabulary of nearly critical modes.

## Materials and Methods

### Experimental Data.

The experimental data used in this study are published fully annotated and with loading and analysis software in a scientific data publication (6). In brief, a macaque monkey is performing a reach-to-grasp task. Activity in the arm-related region of area M1 is recorded using a 10×10-electrode Utah array. T=400 ms after start of the recording, the monkey receives a visual fixation cue to announce the beginning of a trial. Multiple subsequent visual cues indicate grip and force types for the grasp, and after a delay period, the monkey performs the actual movement. In this study, we are solely considering the first T=400 ms of the recording in each trial before the first cue and devoid of task-related behavior. Although the monkey is expecting a trial to begin, this period most closely represents resting state-type activity with stationary spiking statistics (*SI Appendix*).

### Linking Data and Theory of Correlations in Spiking Networks.

**Fig. 8.**
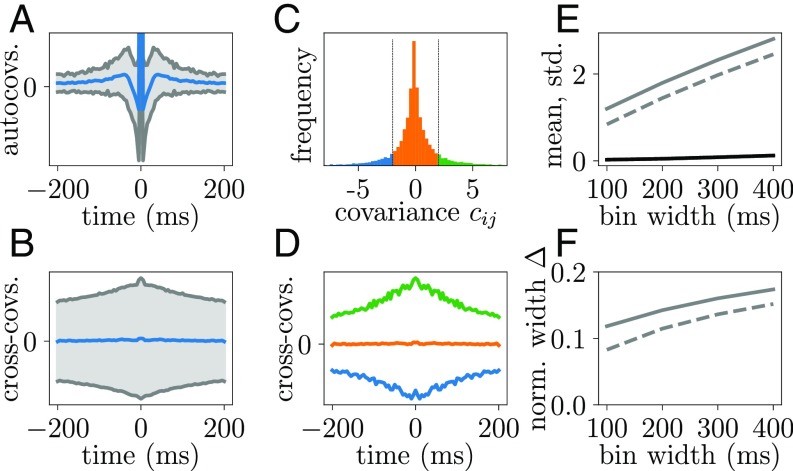
Covariance functions of motor cortex data. (*A*) Mean (blue trace) ± 1 SD (gray traces) of single-neuron autocovariance functions ($t_{bin}=5\;ms$; peak at zero time lag not displayed). (*B*) Mean (blue trace) ± 1 SD (gray traces) of pairwise cross-covariance functions ($t_{bin}=5\;ms$). (*C*) Same spike count covariance histogram as in Fig. 1*D* divided into three ranges (bounds ±2). (*D*) Pairwise cross-covariance functions averaged according to division in *C* shown in corresponding colors. (*E*) Mean (black trace) and SD (solid gray line) of distribution of spike count covariances as a function of bin width. Dashed gray line indicates unbiased estimator of SD (*SI Appendix*). (*F*) Normalized width Δ of covariances (biased estimator: solid curve; unbiased estimator: dashed curve) as a function of bin width. Bin width of 400 ms is used throughout the following analyses.

Spike count covariances $c_{ij}$ are a common measure of neuronal coordination (26). For large observation times T, they are related to the integral of the time-lagged covariance function $c_{ij}\left(\tau \right)$ (34):

$$\begin{array}{ll} c_{ij} & =\frac{1}{T}\left(\left\langle n_{i}n_{j}\right\rangle -\left\langle n_{i}\right\rangle \left\langle n_{j}\right\rangle \right)=\int_{-T}^{T}\frac{T-|\tau |}{T}c_{ij}\left(\tau \right)\;d\tau \\ & \underset{T\to \infty }{\to }\int_{-\infty }^{\infty }c_{ij}\left(\tau \right)\;d\tau , \end{array}$$ [2]

where $n_{i}=\int_{0}^{T}s_{i}\left(t\right)dt$ denotes the spike count of neuron i in the time window [0,T]. Integrated time-lagged covariance functions have been derived for spiking neural networks using linear response theory that reliably captures stationary-state fluctuations (15–17). The applicability of this theory to experimental data, therefore, relies on stationarity of the spiking statistics. The weak fluctuations in population activity in Fig. 1*E* are a first hint that our chosen data segment of the reach-to-grasp experiment fulfills this requirement. More detailed analyses (*SI Appendix*) show nonstationarities in firing rates and correlations but only in later trial segments where the monkey receives visual stimuli or performs movements. The requirement of stationarity, therefore, restricts the observation window to T=400 ms shown in Fig. 1. For such small T, the equivalence between spike count covariances and integrated time-lagged covariance functions does not hold exactly, as the covariance functions do not decay to zero within T (Fig. 8 *A* and *B*). The wide distribution of cross-covariances at large time lags can be investigated by splitting the neuron pairs among three groups based on their spike count covariance (Fig. 8*C*): neuron pairs with a big absolute value for the spike count covariance also have slower decaying time-lagged covariance functions (Fig. 8*D*). The absolute value of the integral of the latter is, therefore, systematically underestimated. As a consequence, the distribution of spike counts depends on the observation time T. In particular, we notice that the width of the distribution increases with increasing window size, while the mean roughly stays around zero on this scale (Fig. 8*E*). We here choose the maximum (defined by stationarity) window T=400 ms and obtain the respective width as a lower bound for the width of the distribution of integrated time-lagged covariance functions that would be measured for T→∞. This choice amounts to a conservative estimation on which we base the results in this study. Given their precise relation exposed above, in the remainder of this manuscript, we refer to both the experimentally measured spike count covariances and the theoretically derived integrated time-lagged covariance functions as covariances.

Uncertainty in the estimation of covariances arises from the limited number of trials. The bias imposed on the width of the distribution of covariances can, however, be computed and corrected for (*SI Appendix*). The finite amount of data prohibits, however, a direct comparison of the shape of the full distributions of covariances between theory and experiment (e.g., via a Kullback–Leibler divergence); an estimation of the bias, as we obtained for the width of the distribution, seems unfeasible for the entire shape of the distribution.

### Mean Field Theory for the Metastatistics of Activity.

Pairwise covariances in the activity of neurons, to a good approximation, follow the simple law (15–17)

$$c\left(W\right)={\left[I-W\right]}^{-1}D{\left[I-W^{T}\right]}^{-1},$$ [3]

which relates the integral of the time-lagged covariances $c_{ij}$ defined by Eq. **2** for T→∞ between all pairs of neurons to the effective connectivity matrix W of the network. I denotes the identity matrix, and the matrix D can be determined from firing rates and shared and correlated external inputs, but the final results of this study turn out to be independent of D. The equation is independent of the neuron model (18), neuronal time constants, and synaptic delays. Therefore, basing our analysis on this fundamental relation guarantees model-independent and robust results.

Starting from Eq. **3**, our aim is to obtain a relation between the statistics of covariances of the activity data and the statistics of connections. The former can be assumed not to depend crucially on the subsampling so that its measurement can be used to obtain a robust prediction for the latter. Although local cortical networks show nonrandom, cell-type specific, and distance-dependent connectivity, the simple model of a homogeneous random network studied in Fig. 3 is sufficient to explain gross features of the experimental data. To calculate the dispersion of covariances, we apply a well-established analytical technique for disordered physical systems: instead of considering all pairwise covariances in a single network, we observe how the covariance of an arbitrary pair of neurons changes for different realizations of the network connectivity. The connectivity appears as an inverse matrix in Eq. **3**, which technically complicates the analysis: no results from random matrix theory apply to this particular problem. Instead, we construct a moment-generating function (58) for the linearized network dynamics (*SI Appendix*), which allows for the use of spin glass techniques (20, 59) combined with approximations for large-N field theories (60). This study is a combination of these individually well-established methods to solve the problem formulated here. As a result, the randomness contained in the $∼N^{2}$ entries of the connectivity formally reduces to only two fluctuating auxiliary variables that provide input to a fully symmetric all-to-all connected effective network. Its high degree of symmetry leads to a drastic reduction of the dimensionality of the equations, which enables us to obtain a mean field theory that describes the neuron-to-neuron variability (*SI Appendix*). This theory yields the mean and SD of variances (i=j) and covariances (i≠j) to leading order in the network size N:

$$c_{ij}={\left[{\left[I-\mu J\right]}^{-1}D_{\lambda }{\left[I-\mu J\right]}^{-1}\right]}_{ij},$$ [4]

$$\delta c_{ij}=\sqrt{\frac{1+\delta_{ij}}{N}\left({\left(\frac{1}{1-\lambda_{\max}^{2}}\right)}^{2}-1\right)}\;D_{\lambda }.$$ [5]

Here, J denotes the matrix of ones, $\mu ∼O\left(1/\sqrt{N}\right)$ is the mean, and $\sigma^{2}=\lambda_{\max}^{2}/N∼O\left(1/N\right)$ is the variance of connection weights in W. The latter determines the radius $\lambda_{\max}$ of the bulk of eigenvalues (Fig. 3*B*) and the renormalized matrix $D_{\lambda }=D/\left(1-\lambda_{\max}^{2}\right)$, which accounts for the structural variability of connections. Eq. **4** predicts that the mean covariances are low [$c_{ij}∼O\left(1/N\right)$] if the network is inhibition dominated (μ<0) (Fig. 5, Case 1) (9, 10). For large spectral radii $\lambda_{\max}≲1$, Eq. **5**, moreover, predicts a large SD [$\delta c_{ij}∼O\left(1/\sqrt{N}\right)$] as experimentally observed in our data (Fig. 1*D*).

When applying the above results to experimental data, the problem arises that $D_{\lambda }$ is not known and cannot be measured. Also, in a spiking network, the autocovariance $c_{ii}$, which is predominantly given by $D_{\lambda }$, is not captured reliably in linear response theory due to spike reset and refractory effects. Both problems can be avoided by considering the ratio $\Delta =\delta c_{ij}/c_{ii}$, which is independent of $D_{\lambda }$ and well predicted by the theory, also for spiking networks (Fig. 4*C*). It is predominantly constrained by the network size and the most unstable eigenvalue, $\lambda_{\max}$, which for inhibition-dominated networks, is determined by the randomness of connectivity arising from its sparsity.

## Supplementary Material

Supplementary File
