# Supplementary material for: Second type of criticality in the brain uncovers rich multiple-neuron dynamics
Source: Proc Natl Acad Sci U S A. 2019 Jun 12;116(26):13051–60. doi: 10.1073/pnas.1818972116 (PMC6600928; doi:10.1073/pnas.1818972116)
Supplement: Supplementary File [file pnas.1818972116.sapp.pdf]

PNAS
www.pnas.org

Supplementary Information for

Second type of criticality in the brain uncovers rich multiple-neuron dynamics

David Dahmen[a,b,c,1], **Sonja Grün**[a,b,c,d], **Markus Diesmann**[a,b,c,e,f], **and Moritz Helias**[a,b,c,f]

[a]Institute of Neuroscience and Medicine (INM-6), Jülich Research Centre, 52425 Jülich, Germany; [b]Institute for Advanced Simulation (IAS-6), Jülich Research Centre, 52425 Jülich, Germany; [c]JARA Institute Brain Structure–Function Relationships (INM-10), Jülich–Aachen Research Alliance, Jülich Research Centre, 52425 Jülich, Germany; [d]Theoretical Systems Neurobiology, RWTH Aachen University, 52056 Aachen, Germany; [e]Department of Psychiatry, Psychotherapy and Psychosomatics, School of Medicine, RWTH Aachen University, 52074 Aachen, Germany; [f]Department of Physics, Faculty 1, RWTH Aachen University, 52062 Aachen, Germany

Corresponding Author: Dahmen
Email: d.dahmen@fz-juelich.de

This PDF file includes:

Supplementary text
Figs. S1 to S7
Table S1
References for SI reference citations

Supporting Information Text

1. Experimental data

Stationarity of experimental data. In this study we apply results from linear response theory for spiking networks to derive a relation between the covariances and the effective connectivity of the network. The resulting expression for the covariance (Eq. 3 in main text) relies on stationarity of the spiking statistics. A priori, one cannot assume such stationarity in an electrophysiological recording in motor cortex subject to a reach-to-grasp protocol. Indeed, over the course of the trial, both firing rate and correlations change (Fig. S1A,C). However, it is possible to identify certain segments of the trial, where both measures are stationary. The first segment is given by the first $T = 400\,\mathrm{ms}$ of the recording (Fig. S1B,D), the period which is used for analysis in this study. This period is devoid of movements and task-related stimuli. A second period ($t \in [700\,\mathrm{ms}, 2000\,\mathrm{ms}]$) also shows stationary statistics, although the monkey receives several visual cues within that time. Therefore this activity is task-related. An analysis of this later segment and quantitative comparison is left for future studies.

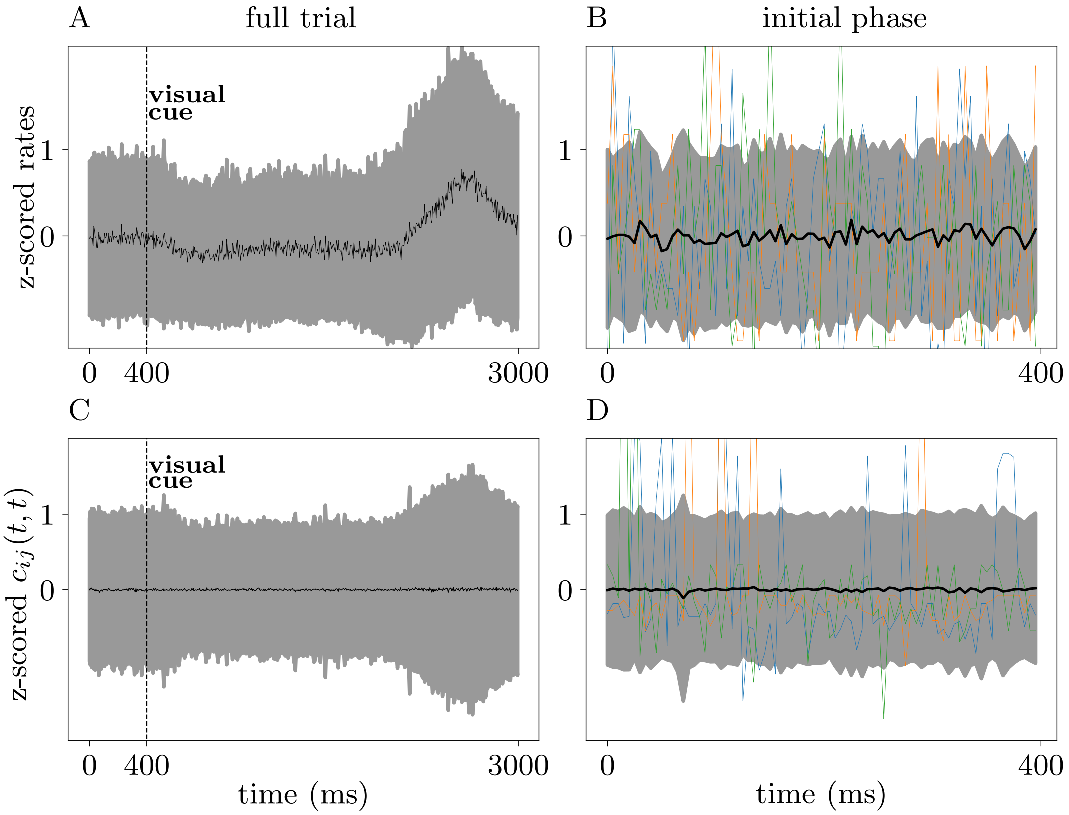

Fig. S1. Stationarity of firing rates and correlations. (**A**) Mean (black trace) plus/minus one standard deviation (gray area) of $z-$scored, time-resolved single-neuron firing rates $r_i(t)$ ($t_{\mathrm{bin}} = 5\,\mathrm{ms}$) for the full trial length of the reach-to-grasp experiment. (**B**) Same as (A), but for first $T = 400\,\mathrm{ms}$ of the trial only. Colored traces show individual neurons. (**C**) Mean (black trace) plus/minus one standard deviation (gray traces) of $z-$scored, time-resolved zero time lag pairwise covariance $c_{ij}(t,t)$ ($t_{\mathrm{bin}} = 5\,\mathrm{ms}$). (**D**) Same as (C), but for first $T = 400\,\mathrm{ms}$ of the trial only. Colored traces show individual neuron pairs. z-score computed with mean and standard deviation over full time window $t \in [0, 3000]\,\mathrm{ms}$.

Bias correction for estimating the width of the distribution of spike count covariances. Due to limited numbers of trials, the estimator for the dispersion of the covariance may be biased towards larger variances. We seek to find a correction for this bias, as illustrated in Fig. S2A. To this end we derive the dependence of experimentally estimated moments of the activities on the number of neurons and trials. The derivation here follows the standard approach of correcting the estimation, sometimes called Bessel's correction (1).

We assume a probability distribution $p(n^1, ..., n^{N_T})$, $n^k \in \mathbb{N}_0^N$, of activities n_i^k of neuron i in trial $k \in \{1, ..., N_T\}$. We assume that the spike counts are sufficiently large so that their statistics is to leading order described by its first two cumulants. We further assume that there are no correlations between different trials, so that the probability distribution factorizes over trials and the moment generating function reads

$$\phi(l^1, ..., l^{N_T}) = \prod_{k=1}^{N_T} \exp\left(m^{\mathrm{T}} l^k + \frac{1}{2} l^{k,\mathrm{T}} c l^k\right), \qquad [1]$$

where m is the vector of mean activities with mean $\overline{m}$ and variance $\overline{\delta m^2}$ across neurons, c is the covariance matrix with mean autocovariance $\overline{a}$, variance of autocovariances $\overline{\delta a^2}$, mean cross-covariance $\overline{c}$ and variance of cross-covariances $\overline{\delta c^2}$ across neurons. The meta-statistics are assumed to be identical across trials. Furthermore, we assume the meta-statistics to be the same for all realizations of distributions of mean activities and covariances across neurons. In the following, $\langle\rangle$ denotes the average over these realizations obtained from the averaged moment generating function

$$\begin{aligned} \left\langle \phi(l^1, ..., l^{N_T}) \right\rangle = {} & e^{\sum_{k=1}^{N_T} \left(\overline{m} \sum_i l_i^k + \frac{1}{2} \overline{a} \sum_i l_i^k l_i^k + \frac{1}{2} \overline{c} \sum_{i \neq j} l_i^k l_j^k \right)} \\ & \cdot e^{\sum_{k,l=1}^{N_T} \left(\frac{1}{2} \overline{\delta m^2} \sum_i l_i^k l_i^l + \frac{1}{8} \overline{\delta a^2} \sum_i l_i^k l_i^k l_i^l l_i^l \right)} \\ & \cdot e^{\sum_{k,l=1}^{N_T} \left(\frac{1}{8} \overline{\delta c^2} \sum_{i \neq j} l_i^k l_j^k l_i^l l_j^l \right)} \end{aligned} \qquad [2]$$

and ˆ denotes the empirical estimates of mean activities and covariances from N recorded neurons in N_T trials of the experiment. Note that the average across realizations formally introduces correlations between different trials (mixed k, l terms in (2)) which allow us to calculate corrections due to the finite number of trials. Using Eq. (2), it is straight-forward to show and well known that an empirical covariance defined as $\hat{c}_{ij}^{\mathrm{b}} = \frac{1}{N_T} \sum_{k=1}^{N_T} (n_i^k - \hat{m}_i)(n_j^k - \hat{m}_j)$ yields a biased estimator:

$$\begin{aligned} \left\langle \hat{c}_{ij}^{\mathrm{b}} \right\rangle &= \frac{1}{N_T} \sum_{k=1}^{N_T} \left\langle (n_i^k - \hat{m}_i)(n_j^k - \hat{m}_j) \right\rangle \\ &= \frac{1}{N_T} \sum_{k=1}^{N_T} \left\langle n_i^k n_j^k \right\rangle - \langle \hat{m}_i \hat{m}_j \rangle \\ &= \frac{1}{N_T} \sum_{k=1}^{N_T} \left\langle n_i^k n_j^k \right\rangle - \frac{1}{N_T^2} \sum_{k,l=1}^{N_T} \left\langle n_i^k n_i^l \right\rangle \\ &= \frac{N_T - 1}{N_T} \left(\delta_{ij} \overline{a} + (1 - \delta_{ij}) \overline{c} \right) \end{aligned} \qquad [3]$$

with $\hat{m}_i = \frac{1}{N_T} \sum_{k=1}^{N_T} n_i^k$ and $\left\langle n_i^k n_j^l \right\rangle = \delta_{ij}(\delta_{kl}\overline{a} + \overline{\delta m^2}) + (1 - \delta_{ij})\delta_{kl}\overline{c} + \overline{m}^2$. The unbiased estimator is therefore given by $\hat{c}_{ij} = \frac{N_T}{N_T - 1} \hat{c}_{ij}^{\mathrm{b}} = \frac{1}{N_T - 1} \sum_{k=1}^{N_T} (n_i^k - \hat{m}_i)(n_j^k - \hat{m}_j)$ (1). Using this definition, along the same lines a lengthy, but straightforward analogous calculation shows that the variance of cross-covariances ($i \neq j$) defined as $\hat{\overline{\delta c^2}} = \frac{1}{N(N-1)} \sum_{i \neq j = 1}^{N} (\hat{c}_{ij} - \hat{\overline{c}})^2$ with the mean cross-covariance across neurons $\hat{\overline{c}} = \frac{1}{N(N-1)} \sum_{i \neq j = 1}^{N} \hat{c}_{ij}$ yields the biased estimator

$$\left\langle \hat{\overline{\delta c^2}} \right\rangle = \overline{\delta c^2} + \frac{\overline{a}^2 - \overline{c}^2}{N_T - 1} \qquad [4]$$

of the true variance of cross-covariances $\overline{\delta c^2}$. Here, we omitted terms of order $\mathcal{O}(1/N^2)$ and $\mathcal{O}(1/(N \cdot N_T))$ which are negligible for the numbers of neurons and trials considered here. For a finite number of trials, there is a significant bias of $\left\langle \hat{\overline{\delta c^2}} \right\rangle$ caused primarily by the average variance $\overline{a}$ of spike-counts across trials (see Fig. S2A for an empirical estimate of the bias with trial-shuffled data).

The aim is hence to correct for the bias of the estimator. This can be formally done using Eq. (4), which we validate for synthetic data in Fig. S2B. A fit of Eq. (4) to the variance of cross-covariances obtained from subsampled numbers of trials of the experimental data shows that the true variance of cross-covariances can be approximately obtained by subtracting the mean of the distribution of the surrogate data in Fig. S2A.

In conclusion, the finite number of trials yields a significant bias to the variance of cross-covariances, but not to the mean autocovariances, if properly defined (see Eq. (3)). Fitting data to Eq. (4) and extrapolating for $N_T \to \infty$ yields the unbiased

estimate which we use in the main text. However, even neglecting this correction, the order of magnitude of the ratio between the two estimates, which determines the largest eigenvalue (Eq. 1 in the main text) and the operational regime, is not changed by the bias.

In addition to the bias in the estimation of the variance, the estimator comes with a statistical uncertainty due to the limited number of recorded neurons. We observe $n = N(N-1)$ cross-covariances of which we want to estimate the true variance. The relative standard error of the variance is therefore given by $\sqrt{2/(n-1)} \approx 0.009 < 1\%$ (2). Error propagation to the estimation of $\lambda^2_{\max}$ shows that the relative error of $\lambda^2_{\max}$ is even smaller for $\lambda^2_{\max} > 1/3$, and hence in the critical regime close to $\lambda^2_{\max} \simeq 1$ this error is negligible.

Qualitatively similar distributions of correlations have been obtained for other cortical areas, e.g. in visual cortex of macaque (3). In this study, the authors consider correlations coefficients $\hat{z}_{ij}$ rather than covariances $\hat{c}_{ij}$, and showed that mean correlations are close to zero. Furthermore, they showed a substantial contribution to the dispersion of correlation coefficients arising from finite data (see Fig. S3 in (3)). The remaining variance after this bias correction can be compared to our data if we re-interpret the spike counts n_i^k in the above calculations by the normalized spike counts $n_i^k \to \frac{n_i^k}{\sqrt{\hat{c}_{ii}}}$. Then the same derivation as above holds and we obtain an expression for the width of the biased estimator of the variance $\widehat{\overline{\delta z^2}}$ of correlation coefficients in terms of the true variance $\overline{\delta z^2}$ of correlation coefficients

$$\left\langle \widehat{\overline{\delta z^2}} \right\rangle = \overline{\delta z^2} + \frac{1 - \overline{z}^2}{N_T - 1}. \qquad [5]$$

For macaque motor cortex, we obtain a mean correlation coefficient $\overline{z} = 0.007$ and a standard deviation $\delta z_{ij} = \sqrt{\overline{\delta z^2}} = 0.10$. Both values are on the same order of magnitude as in (3) ($\overline{z} = 0.01$, $\delta z_{ij} = 0.06$), but the dispersion is larger in motor cortex. This motivates more detailed future investigations of the distributions of correlations in various areas in relation to their effective network size.

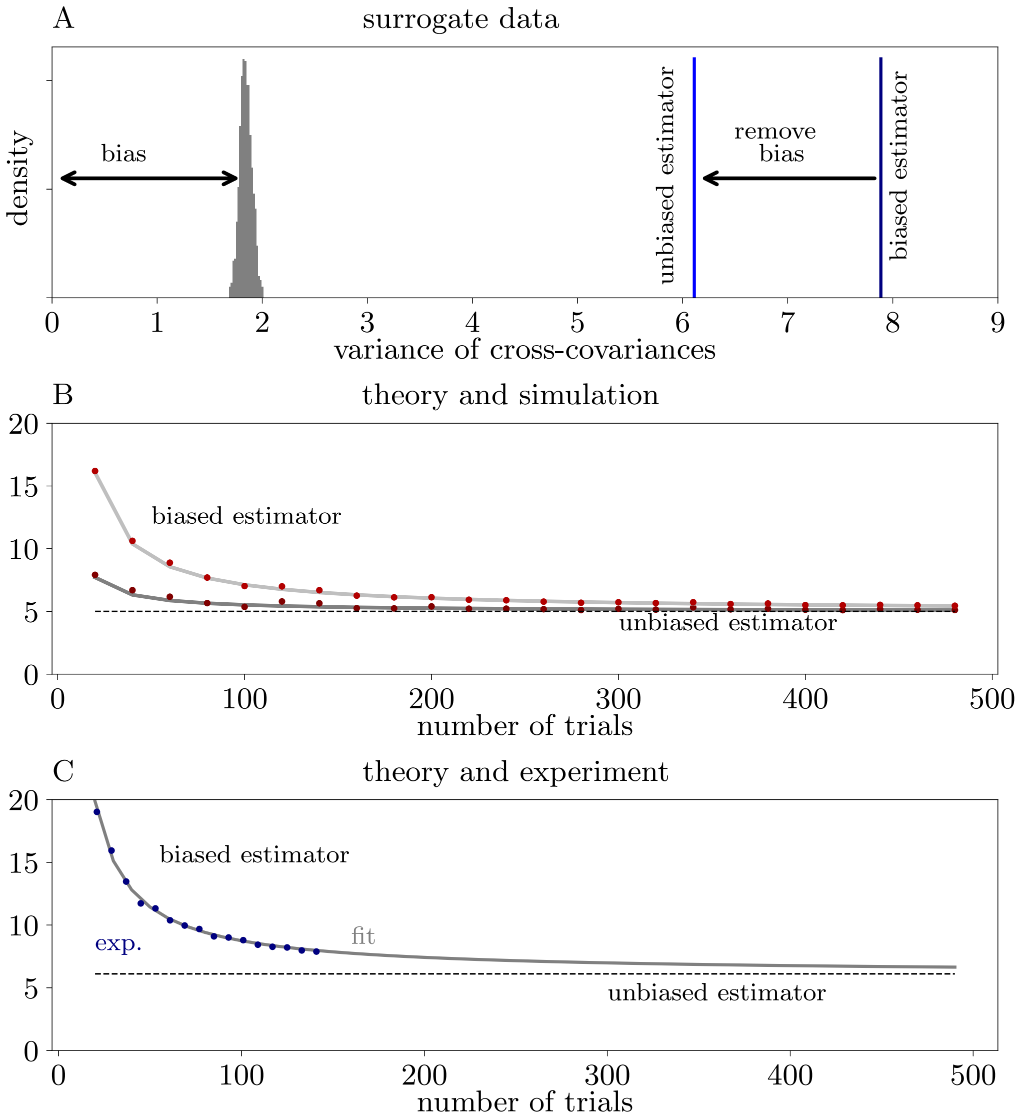

Fig. S2. Assessment and correction of the bias in estimating variance of cross-covariances. (**A**) Variance of cross-covariances (gray distribution) obtained from trial-shuffled data compared to the biased estimator (equation Eq. (4), dark blue) and unbiased estimator (light blue) of the true variance as obtained from experimental data. (**B**) Convergence of biased estimator for small (dark gray) and large (light gray) mean covariances towards true variance given by unbiased estimator (dashed line). Curves indicate the theoretical prediction equation Eq. (4), dots numerical simulations for artificial data. (**C**) True value 6.11 of variance of cross-covariances (dashed line) derived from fitting (gray curve) equation Eq. (4) to the biased estimator (blue dots) obtained from subsampled experimental data with different numbers of trials as given by the abscissa.

2. Mean-field theory for meta-statistics of activity

Mean-field theory most-commonly employs the thermodynamic limit ($N \to \infty$), reducing the collective dynamics of the N interacting units to N pairwise independent units, each subject to a self-consistently determined auxiliary field (4–9). Covariances of individual neurons are self-averaging in this limit, so they are identical for all units and independent of the realization of the randomness in the network connectivity. In particular, cross-covariances vanish in the limit. In the absence of external stimuli and in the weakly correlated regime, covariances can be understood in linear response theory (10). For such a linearized network model, interactions between neurons can be included as a finite-size correction within this self-averaging framework (11–15). While this procedure yields covariances averaged over many pairs of units, the experimentally observed neuron to neuron variability (3) is lost. Here, we develop a theory beyond self-averaging covariances that captures their statistics. It exploits the large size of biologically realistic local networks, but includes finite-size fluctuations of auxiliary fields which derive from the quenched disorder of the couplings to explain the variability of covariances. To perform this qualitative step, we need to combine methods from different fields: We use a functional formulation of Gaussian processes that originates from statistical field theory (16–18) and combine it with methods typically used for disordered systems in the large N limit (19), such as spin glasses (20, 21).

Moment generating functional for the network dynamics. In the following, we consider time-lag integrated covariances

$$c_{ij} = \int c_{ij}(\tau) d\tau = \int \int \langle x_i(t+\tau) x_j(t) \rangle_x \, dt \, d\tau$$
$$= \langle X_i(0) X_j(0) \rangle_x \qquad [6]$$

calculated as averages $\langle\rangle_x$ across different trials of the linearized network dynamics $x(t)$ or its Fourier transform $X(\omega)$ evaluated at $\omega = 0$ (Wiener-Khinchin theorem, (22, sec. 1.4.2)). The linearized dynamics can be modeled as a set of coupled Ornstein-Uhlenbeck processes

$$\tau \frac{dx(t)}{dt} = -x(t) + W \cdot x(t) + \xi(t) \qquad [7]$$

with the moment-generating functional

$$Z[j] = \int \mathcal{D}x \int \mathcal{D}\tilde{x} \, \exp\left(S_0[x, \tilde{x}] + j^{\mathrm{T}} x \right)$$
$$\text{with} \quad S_0[x, \tilde{x}] = \tilde{x}^{\mathrm{T}} \left((\partial_t + 1)\mathcal{I} - W \right) x + \frac{D}{2} \tilde{x}^{\mathrm{T}} \tilde{x}. \qquad [8]$$

Here, $\mathcal{I}$ denotes the identity matrix. Here $\xi(t)$ is a Gaussian white noise with variance $\langle \xi_i(t) \xi_j(t') \rangle = D \delta_{ij} \delta(t - t')$, $\tilde{x}(t)$ is a purely imaginary response field, $\int \mathcal{D}\tilde{x}$, $\int \mathcal{D}x$ are suitably defined path integral measures, and $\tilde{x}^{\mathrm{T}} \tilde{x} = \sum_i \int \tilde{x}_i(t) \, \tilde{x}_i(t) \, dt$ is a scalar product (16–18). The generating functional can easily be interpreted in Fourier domain due to the linearity of equation Eq. (7) and the invariance of scalar products under unitary transforms

$$Z[J] = \int \mathcal{D}X \int \mathcal{D}\tilde{X} \, \exp\left(S_0[X, \tilde{X}] + J^{\mathrm{T}} X \right)$$
$$\text{with } S_0[X, \tilde{X}] = \tilde{X}^{\mathrm{T}} \left((i\omega + 1)\mathcal{I} - W \right) X + \frac{D}{2} \tilde{X}^{\mathrm{T}} \tilde{X}, \qquad [9]$$

with Fourier transformed variables denoted by capital letters. The scalar product in frequency domain reads $\tilde{X}^{\mathrm{T}} X = \sum_i \int \tilde{X}_i(-\omega) X_i(\omega) \, d\omega$. The generating functional factorizes into generating functions for each frequency ω. As shown in Eq. (6), time-lag integrated covariances only require the knowledge of $X(0)$. In the following, we will therefore only discuss zero frequency. After integration over all non-zero frequencies one obtains the generating function for zero frequency

$$Z(J) = \det(1 - W) \int \mathcal{D}X \int \mathcal{D}\tilde{X} \, e^{S_0(X, \tilde{X}) + J^{\mathrm{T}} X} \qquad [10]$$
$$= \exp\left(\frac{1}{2} J^{\mathrm{T}} \left(\mathcal{I} - W \right)^{-1} D \left(\mathcal{I} - W^{\mathrm{T}} \right)^{-1} J \right)$$
$$S_0(X, \tilde{X}) = \tilde{X}^{\mathrm{T}} \left(\mathcal{I} - W \right) X + \frac{D}{2} \tilde{X}^{\mathrm{T}} \tilde{X}, \qquad [11]$$

with the single-frequency ($\omega = 0$) scalar product defined as $\tilde{X}^{\mathrm{T}} X = \sum_i \tilde{X}_i X_i$, and integration measures $\int \mathcal{D}X = \prod_j \int_{-\infty}^{\infty} dX_j$ and $\int \mathcal{D}\tilde{X} = \prod_j \frac{1}{2\pi i} \int_{-i\infty}^{i\infty} d\tilde{X}_j$. The determinant in Eq. (10) follows from the normalization condition $Z(J = 0) = 1$. The time-lag integral of the covariance functions follows as

$$c(W) = \left[\mathcal{I} - W \right]^{-1} D \left[\mathcal{I} - W^{\mathrm{T}} \right]^{-1}, \qquad [12]$$

in line with Eq. 3 in the main text.

Self-averaging meta-statistics. Equation (12) relates covariances between individual pairs of neurons to the connectivity matrix W. These, however, change between realizations of the random connectivity. In contrast, the meta-statistics, by which we denote the moments of the distribution of covariances, can be assumed constant across realizations (self-averaging, (21)). We therefore seek for an expression relating the moments of the distribution of covariances to the statistics of the connectivity W.

We denote with $\bar{\ }$ the empirical average, with $\langle\rangle_x$ the expectation over realizations of the processes x, and with $\langle\rangle$ the ensemble average over the disordered connectivity W. Exchanging the order of differentiation and averaging allows expressing second moments of covariances as derivatives of a single disorder-averaged generating function $\langle Z(J)\rangle$: We first assume the empirical average to be self-averaging

$$\overline{c_{ii}^2} = \left\langle \overline{c_{ii}^2} \right\rangle . \qquad [13]$$

We then use its definition as $\overline{c_{ii}^2} = \frac{1}{N}\sum_{i=1}^{N} c_{ii}^2$, and that of the second cumulant $c_{ij} = \langle X_i X_j \rangle_x$ of the zero frequency components $X = X(\omega = 0) = \int_{-\infty}^{\infty} x(t)\, dt$ of Ornstein-Uhlenbeck processes x to obtain

$$\overline{c_{ii}^2} = \frac{1}{N}\sum_{i=1}^{N} \left\langle \langle X_i X_i \rangle_x^2 \right\rangle . \qquad [14]$$

As the action (10) for a single realization of W is quadratic, Wick's theorem applies, $\langle X_i X_i X_j X_j \rangle_x = 2\langle X_i X_j \rangle_x^2 + \langle X_i X_i \rangle_x \langle X_j X_j \rangle_x = 2c_{ij}^2 + c_{ii}c_{jj}$ for the special case $i = j$, such that we may identify the squared second moment (14) with a fourth moment. The latter can be expressed with the help of the generating function

$$\begin{aligned} \overline{c_{ii}^2} &= \frac{1}{N}\sum_{i=1}^{N} \left\langle \langle X_i X_i \rangle_x^2 \right\rangle \overset{\text{Wick's th.}}{=} \frac{1}{N}\sum_{i=1}^{N} \frac{1}{3} \left\langle \langle X_i X_i X_i X_i \rangle_x \right\rangle \\ &= \frac{1}{N}\sum_{i=1}^{N} \frac{1}{3} \left\langle \frac{d^4}{dJ_i^4} Z(J) \right\rangle \Bigg|_{J=0} = \frac{1}{3}\, \frac{d^4}{dJ_i^4} \langle Z(J)\rangle \Bigg|_{J=0} . \end{aligned} \qquad [15]$$

In the last step, we exchanged the order of derivatives and the expectation value over network realizations and used the symmetry of the disorder-averaged network over units. Analogously follows for $i \neq j$ and $N - 1 \approx N$,

$$\overline{c_{ij}^2} = \frac{1}{2}\, \frac{d^4}{dJ_i^2 dJ_j^2} \langle Z(J)\rangle \Bigg|_{J=0} - \frac{1}{2} \langle \overline{c_{ii}} \rangle^2 . \qquad [16]$$

Disorder-averaged generating function. Ignoring insignificant variations in the normalization $\det(1 - W)$ of $Z(J)$, the disorder average only affects the coupling term in Eq. (10)

$$\left\langle e^{\tilde{X}^{\mathrm{T}} W X} \right\rangle = \prod_{i,j} \left\langle e^{W_{ij} \tilde{X}_i X_j} \right\rangle = \prod_{i,j} e^{\sum_{k=1}^{\infty} \frac{\kappa_k}{k!} (\tilde{X}_i X_j)^k} .$$

For clarity, we here assume independent and identically distributed weights W_{ij}. In the resulting cumulant expansion (21, 23–25) κ_k is the k-th cumulant for a single connection W_{ij} (26). For fixed connection probability p, the number of inputs to a neuron scales with the network size N. To keep the input and its fluctuations within a certain dynamic range when increasing the network size, we require synaptic weights to scale with $1/\sqrt{N}$ (5, 27), such that the cumulant expansion is an expansion in $1/\sqrt{N}$. A truncation at the second cumulant ($\propto N^{-1}$) maps W to a Gaussian connectivity $\mathcal{N}(\mu, \lambda_{\mathrm{max}}^2/N)$ so that

$$\begin{aligned} \langle Z(J)\rangle &\sim \int \mathcal{D}X \int \mathcal{D}\tilde{X}\, e^{S_0(X,\tilde{X}) + \frac{\lambda_{\mathrm{max}}^2}{2N} V(X,\tilde{X}) + J^{\mathrm{T}} X} , \qquad [17] \\ S_0(X,\tilde{X}) &= \tilde{X}^{\mathrm{T}} \left(\mathcal{I} - \mu\mathcal{J}\right) X + \frac{D}{2} \tilde{X}^{\mathrm{T}} \tilde{X} , \\ V(X,\tilde{X}) &= \tilde{X}^{\mathrm{T}} \tilde{X}\, X^{\mathrm{T}} X \end{aligned}$$

with a homogeneous mean connection weight $\mu = \mathcal{O}(1/\sqrt{N})$. Here, $\mathcal{J}$ denotes the matrix of ones. The second cumulant ($\lambda_{\mathrm{max}}^2/N$) is the first non-trivial contribution to the second moment of covariances. While higher cumulants of the connectivity have an impact on higher moments of the distribution of covariances, their effect on the first two moments is suppressed by the large network size.

Auxiliary-field formalism. The interaction term V prevents an exact calculation of the disorder-averaged generating function. A converging perturbation series can be obtained in the auxiliary-field formulation (19), where a field $Q_1 = \frac{\lambda_{\mathrm{max}}^2}{N} X^{\mathrm{T}} X$ is introduced for the sum of a large number of statistically equivalent activity variables. Using the Hubbard-Stratonovich transformation

$$\begin{aligned} e^{\frac{\lambda_{\mathrm{max}}^2}{2N} \tilde{X}^{\mathrm{T}} \tilde{X}\, X^{\mathrm{T}} X} &= \int_{-\infty}^{\infty} dQ_1 \delta \left(Q_1 - \frac{\lambda_{\mathrm{max}}^2}{N} X^{\mathrm{T}} X \right) e^{\frac{1}{2} Q_1 \tilde{X}^{\mathrm{T}} \tilde{X}} \\ &= \int \mathcal{D}Q e^{-\frac{N}{\lambda_{\mathrm{max}}^2} Q_1 Q_2 + \frac{1}{2} Q_1 \tilde{X}^{\mathrm{T}} \tilde{X} + Q_2 X^{\mathrm{T}} X} , \end{aligned}$$

with $\int \mathcal{D}Q = \frac{1}{2\pi i} \frac{N}{\lambda_{\max}^2} \int_{-\infty}^{\infty} dQ_1 \int_{-i\infty}^{i\infty} dQ_2$ one obtains a free theory, which is a quadratic action in the activity (X) and response variables $(\tilde{X})$, on the background of fluctuating fields Q

$$\begin{aligned} \langle Z(J) \rangle &\sim \int \mathcal{D}Q \, \exp\left(-\frac{N}{\lambda_{\max}^2} Q_1 Q_2 + \ln\left(Z_Q(J)\right)\right), \\ Z_Q(J) &= \int \mathcal{D}X \int \mathcal{D}\tilde{X} \, e^{S_0(X,\tilde{X}) + \frac{1}{2} Q_1 \tilde{X}^{\mathrm{T}} \tilde{X} + Q_2 X^{\mathrm{T}} X + J^{\mathrm{T}} X}. \end{aligned} \quad [18]$$

The high dimensional integrals of the free theory $Z_Q(J)$ can be solved analytically yielding a two-dimensional interacting theory in the auxiliary fields Q_1 and Q_2. The auxiliary field formalism translates the high-dimensional ensemble average over W to a low-dimensional average over Q; it maps the local disorder in the connections to fluctuations of global fields Q interacting with a highly symmetric all-to-all connected network, illustrated in Fig. S3. Only in the special case of vanishing mean connection strength $\mu = 0$, the system factorizes into N unconnected units, each interacting with the same set of fields Q. The all-to-all network not only captures the autocovariance of a single neuron, but also the cross-covariance with any other neuron.

Saddle-point approximation. Performing a change of variables $Q = Q^*(J) + \frac{\delta Q}{\sqrt{N}}$ in Eq. (18), where $Q^*(J)$ are the saddle-points of the exponent $-\frac{N}{\lambda_{\max}^2} Q_1 Q_2 + \ln\left(Z_Q(J)\right) =: -N \cdot Y(Q_1, Q_2, J)$ determined by the saddle-point equations

$$0 = \frac{\partial}{\partial Q_\alpha} Y(Q_1, Q_2, J)\bigg|_{Q=Q^*(J)} \qquad \alpha \in \{1, 2\}, \quad [19]$$

and expanding $Y(Q_1, Q_2, J)$ around $Q^*(J)$, yields an expansion in powers of $1/N$ (19, 28). We focus only on the leading-order contribution $Y(Q_1^*(J), Q_2^*(J), J)$ which describes tree-level diagrams in the Q-theory and drop all source dependence in higher Taylor coefficients

$$\langle Z(J) \rangle \sim \exp\left(-N \cdot Y(Q_1^*(J), Q_2^*(J), J)\right), \quad [20]$$

with the corresponding action

$$\begin{aligned} S_{Q_\alpha^*(J)}(X, \tilde{X}) = \tilde{X}^{\mathrm{T}} \left(\mathcal{I} - \mu \mathcal{J}\right) X + \frac{D + Q_1^*(J)}{2} \tilde{X}^{\mathrm{T}} \tilde{X} \\ + Q_2^*(J) \, X^{\mathrm{T}} X. \end{aligned} \quad [21]$$

Note that this Gaussian theory in $X, \tilde{X}$ still contains in the latter two terms contributions from the quartic interaction term V of the original theory. Variability in $Q^*(J)$, through their source dependence, therefore gives rise to cumulants of the activity variables beyond second order. The dependence of $Q^*(J)$ on external sources J was neglected in prior work (20) since it scales to leading order as $1/N$. This approximation, however, yields a Gaussian theory (see Eq. (21) for $J = 0$), which does not generate fourth cumulants and therefore no distribution of covariances. Taking into account the J dependence of auxiliary fields in combination with the relation between fourth cumulants and distributions of covariances (15) and (16) thus extends mean-field theory beyond self-averaging covariances.

The action (21) shows that $Q_1^*(J)$ acts as a global contribution to the Gaussian noise whereas $Q_2^*(J)$ directly contributes to the inverse of the covariance matrix. By integrating out the response variables $\tilde{X}$, one can alternatively interpret both $Q_\alpha{}^*(J)$ as contributions to the covariance matrix of the noise.

Saddle points $Q^*(J)$ are obtained self-consistently from (19) which reduces to the set of equations

$$\begin{aligned} Q_1^*(J) &= \frac{\lambda_{\max}^2}{N} \left\langle X^{\mathrm{T}} X \right\rangle_{Q_\alpha^*(J)}(J) \\ Q_2^*(J) &= \frac{\lambda_{\max}^2}{2N} \left\langle \tilde{X}^{\mathrm{T}} \tilde{X} \right\rangle_{Q_\alpha^*(J)}(J) \\ &\text{with} \\ \langle \circ \rangle_{Q_\alpha^*(J)}(J) &:= \frac{\int \mathcal{D}X \int \mathcal{D}\tilde{X} \, \circ \, \exp\left(S_{Q_\alpha^*(J)}(X, \tilde{X}) + J^{\mathrm{T}} X\right)}{\int \mathcal{D}X \int \mathcal{D}\tilde{X} \, \exp\left(S_{Q_\alpha^*(J)}(X, \tilde{X}) + J^{\mathrm{T}} X\right)} \end{aligned} \quad [22]$$

The above set of equations cannot be solved analytically for $Q^*(J)$. However, moments of activity result from derivatives of $\langle Z(J) \rangle$ evaluated at $J = 0$. The derivatives act not only on the source term $J^{\mathrm{T}} X$, but also on the J-dependence of the saddle-point. Therefore, moments are determined by saddle-points and their derivatives evaluated at zero source. Setting $J = 0$ in (22) yields the self-consistent result

$$\begin{aligned} \left\langle \tilde{X}_i \tilde{X}_j \right\rangle_{Q_\alpha^*} &= 0, \\ \left\langle X_i X_j \right\rangle_{Q_\alpha^*} &= \left[\left(\mathcal{I} - \mu \mathcal{J}\right)^{-1} \left(D + Q_1^*\right) \left(\mathcal{I} - \mu \mathcal{J}\right)^{-1} \right]_{ij}, \\ \left\langle \tilde{X}_i X_j \right\rangle_{Q_\alpha^*} &= \left[\left(\mathcal{I} - \mu \mathcal{J}\right)^{-1} \right]_{ij}, \end{aligned} \quad [23]$$

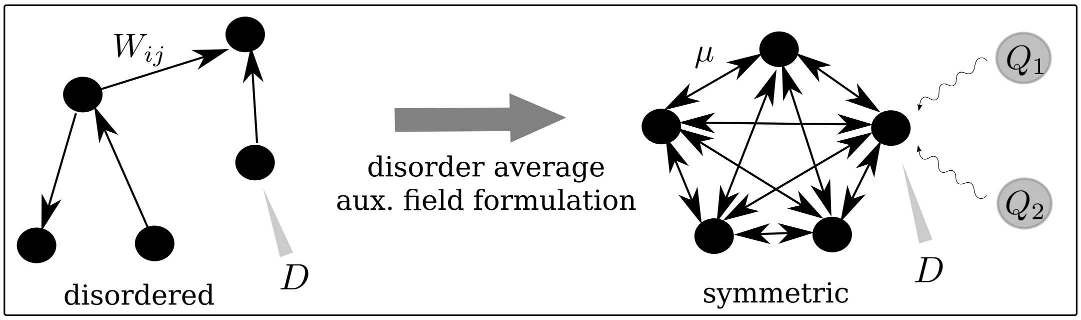

Fig. S3. Reduction of a disordered to a regular network. Disorder average maps network with frozen variability in connections (left) to highly symmetric network on the background of fluctuating auxiliary fields Q (right). Their fluctuations contribute globally to the covariance that drives the network fluctuations (illustrated by wavy arrows).

with

$$Q_1^* := Q_1^*(0) = \frac{R^2}{1-R^2}\,D, \qquad [24]$$
$$Q_2^* := Q_2^*(0) = 0,$$

$\langle\circ\rangle_{Q_\alpha^*} := \langle\circ\rangle_{Q_\alpha^*(0)}(0)$, $R = \sqrt{1+\gamma}\,\lambda_{\max} \approx \lambda_{\max}$ and $\gamma = \mathcal{O}(1/N)$ resulting from subleading terms in (23) containing μ. Equation (23) shows that Q_1^* leads to a renormalization of the noise D as $D_\lambda = D + Q_1^* = D/(1-R^2) \approx D/(1-\lambda_{\max}^2)$.

Analogously, second derivatives of saddle-points with respect to sources can be calculated from (22) with the final result

$$\begin{aligned}
\left.\frac{d^2Q_1^*(J)}{dJ_k dJ_l}\right|_{J=0} &= \frac{\lambda_{\max}^2}{\beta N}\sum_i \langle X_i X_k\rangle_{Q_\alpha^*}\langle X_i X_l\rangle_{Q_\alpha^*} \\
&\quad + \frac{\lambda_{\max}^2}{\beta N}\sum_{i,a}\langle X_i X_a\rangle_{Q_\alpha^*}^2 \left.\frac{d^2Q_2^*(J)}{dJ_k dJ_l}\right|_{J=0}, \\
\left.\frac{d^2Q_2^*(J)}{dJ_k dJ_l}\right|_{J=0} &= \frac{\lambda_{\max}^2}{\beta N}\sum_i \left\langle \tilde{X}_i X_k\right\rangle_{Q_\alpha^*}\left\langle \tilde{X}_i X_l\right\rangle_{Q_\alpha^*}, \\
&\quad \text{with } \beta = 1 - \frac{\lambda_{\max}^2}{N}\sum_{i,a}\left\langle X_i\tilde{X}_a\right\rangle_{Q_\alpha^*}^2,
\end{aligned}$$

where we used that $\left.\frac{dQ_\alpha^*(J)}{dJ_k}\right|_{J=0} = 0$ since three-point correlators vanish.

Using partial derivative calculus $\frac{d}{dJ_i}\langle Z(J)\rangle = \left(\frac{\partial Q_1^*}{\partial J_i}\frac{\partial}{\partial Q_1^*} + \frac{\partial Q_2^*}{\partial J_i}\frac{\partial}{\partial Q_2^*} + \frac{\partial}{\partial J_i}\right)\langle Z(Q_1^*, Q_2^*, J)\rangle$ and the saddle-point condition $\frac{\partial}{\partial Q_\alpha^*}\langle Z(Q_1^*, Q_2^*, J)\rangle = 0$ which follows from (19) and (20), we find that second moments of activity, the average covariances, are not influenced by the source dependence of saddle points

$$\begin{aligned}
\left\langle \langle X_i X_j\rangle_x\right\rangle &= \left.\frac{d^2}{dJ_i dJ_j}\langle Z(J)\rangle\right|_{J=0} = \langle X_i X_j\rangle_{Q_\alpha^*} \\
&= \left[(\mathcal{I}-\mu\mathcal{J})^{-1} D_\lambda (\mathcal{I}-\mu\mathcal{J})^{-1}\right]_{ij}.
\end{aligned} \qquad [25]$$

The non-zero mean connection strength $\mu = \mathcal{O}(1/\sqrt{N})$ yields cross-covariances between neurons due to the finite size of the network. In addition, the fourth moments of activity are crucial for the non-vanishing variance of covariances: Formally, two of the four derivatives act on $Q^*(J)$ while the other two act on the source term $J^{\mathrm{T}}X$ to yield

$$\begin{aligned}
\langle\langle X_i X_j X_i X_j\rangle_x\rangle &= \left.\frac{d^4}{dJ_i dJ_j dJ_i dJ_j}\langle Z(J)\rangle\right|_{J=0} \\
&= 4\left.\frac{d^2Q_1^*(J)}{dJ_i dJ_j}\right|_{J=0}\sum_a \left\langle X_i\tilde{X}_a\right\rangle_{Q_\alpha^*}\left\langle X_j\tilde{X}_a\right\rangle_{Q_\alpha^*} \\
&+ 2\left.\frac{d^2Q_1^*(J)}{dJ_i dJ_i}\right|_{J=0}\sum_a \left\langle X_j\tilde{X}_a\right\rangle_{Q_\alpha^*}\left\langle X_j\tilde{X}_a\right\rangle_{Q_\alpha^*} \\
&+ 4\left.\frac{d^2Q_2^*(J)}{dJ_i dJ_j}\right|_{J=0}\sum_a \langle X_i X_a\rangle_{Q_\alpha^*}\langle X_j X_a\rangle_{Q_\alpha^*} \\
&+ 2\left.\frac{d^2Q_2^*(J)}{dJ_i dJ_i}\right|_{J=0}\sum_a \langle X_j X_a\rangle_{Q_\alpha^*}\langle X_j X_a\rangle_{Q_\alpha^*} \\
&+ 2\langle X_i X_j\rangle_{Q_\alpha^*}\langle X_i X_j\rangle_{Q_\alpha^*} \\
&+ \langle X_i X_i\rangle_{Q_\alpha^*}\langle X_j X_j\rangle_{Q_\alpha^*}.
\end{aligned} \qquad [26]$$

The latter two terms in Eq. (26) correspond to the trivial Wick decomposition from the Gaussian part of the theory at $Q_\alpha^* = Q_\alpha^*(J=0)$, whereas the second derivatives of saddle points $\left.\frac{d^2Q_\alpha^*(J)}{dJ_i dJ_j}\right|_{J=0} = \mathcal{O}(1/N)$ evaluated at zero source determine the non-vanishing fourth cumulants. Note that no quartic derivatives of saddle points appear in Eq. (26) due to the saddle-point condition $\frac{\partial}{\partial Q_\alpha^*}\langle Z(Q_1^*, Q_2^*, J)\rangle = 0$.

Mean and variance of the covariance distribution. We obtain the mean integral covariances (see Eq. (25))

$$\overline{c_{ij}} = \left[(\mathcal{I} - \mu\mathcal{J})^{-1} D_\lambda (\mathcal{I} - \mu\mathcal{J})^{-1}\right]_{ij} = D_\lambda \gamma_{ij} \qquad [27]$$

and the variance of integral covariances (see Eqs. (15), (16) and (26))

$$\overline{\delta c_{ij}^2} = \lambda_{\max}^2 \left[\frac{1}{(1-\lambda_{\max}^2)^2} + \frac{1}{1-\lambda_{\max}^2}\right] D_\lambda^2 \chi_{ij}. \qquad [28]$$

The mean connectivity μ enters the coefficients $\gamma_{ij} = \delta_{ij} + \gamma$ (with δ_{ij} the Kronecker symbol), $\chi_{ij} = \frac{1}{N}(1 + \delta_{ij} + \mathcal{O}(1/N))$ and $\gamma = \frac{\mu}{1-\mu N} = \mathcal{O}(1/N)$ only in their sub-leading corrections, and acts as a negative feedback in inhibitory or inhibition-dominated networks (14). While this feedback suppresses mean cross-covariances (27) which consequently scale as $\overline{c_{ij}} \sim \frac{1}{N}$, it only yields a subleading contribution to the dispersion (28). The spread of individual cross-covariances is determined by fluctuations in connection weights and shows a scaling as $\sqrt{\overline{\delta c_{ij}^2}} \sim \frac{1}{\sqrt{N}}$. These fluctuations, which formally originate from the variability of the auxiliary fields Q, are therefore much larger than the mean; they cause broad distributions of cross-covariances of both signs even in a homogeneous network. The expressions therefore explain the first two moments of the experimentally observed distribution of cross-covariances: mean cross-covariances scale as $\mathcal{O}(1/N)$, whereas the standard deviation only scales as $\mathcal{O}(1/\sqrt{N})$. The width of the distribution is thus much larger than the mean for large networks and the distribution is centered approximately around zero.

We note that this approach is inherently different from mean-field theories for single realizations of network connectivities which keep the site dependence to infer relations between covariances and connections on the level of individual neurons (29). In contrast, we derive a relation between the statistics of the structure and the statistics of the dynamics using a quenched average. The crucial feature of the presented theory is that heterogeneity across neurons can still be extracted from the ensemble description. We here show that the heterogeneity is closely linked to fluctuations of the auxiliary fields, which are accessible by studying their source dependence.

The result (28) relies on the approximation of setting $\gamma \approx 0$, which is almost exact in the dynamically balanced state, where $\mu < 0$. For excitation-dominated networks, an instability can arise for $\mu \to 1/N$, which is neglected here. The theoretical results presented here are thus only applicable to dynamically balanced networks. Furthermore, the single-neuron dynamics must not exhibit strong burstiness as this hinders the applicability of linear response theory.

Nevertheless for excitation-dominated networks away from the limit $\mu \to 1/N$ the theory makes predictions for the scale of the covariances. For stability reasons, these networks require a scaling of weights with $1/N$, so that mean and variance of the weight distribution scale as

$$\mu = pw = \mathcal{O}(1/N),$$
$$\lambda_{\max}^2/N = p(1-p)w^2 = \mathcal{O}(1/N^2).$$

As a consequence the largest bulk eigenvalue $\lambda_{\max}$ scales as $\mathcal{O}(1/\sqrt{N})$. From equations (27) and (28) therefore follows that excitation-dominated networks exhibit distributions of covariances where the mean and standard deviation are on the same order of magnitude $\mathcal{O}(1/N)$ (see Fig. 6G in the main text).

3. Other sources of variability

There are many sources of neuron and circuit heterogeneities that could potentially affect the distribution of covariances and its width. For simplicity, these sources have not been addressed in the theoretical calculations that assume a random network with homogeneous connection statistics and uncorrelated external input. While we already considered networks of excitatory and inhibitory neurons that follow Dale's law and networks with spatially dependent connectivity in Fig. 4 of the main text, in the following we expand this analysis towards a heavy tailed distribution of synaptic weights, a distribution of firing rates, correlated external input, and connection motifs. We show that a large Δ (after bias correction) can only arise from close-to-unstable local dynamics in a multitude of directions in N-dimensional space, thus demonstrating the robustness of our main conclusion with regard to the various sources of heterogeneity.

Effect of log-normal synaptic weight distribution. The theoretical results for the mean and width of the distribution of covariances strictly hold for a Gaussian connectivity matrix. In the process of approximations, any connectivity statistics is mapped to a corresponding Gaussian distribution by truncating the expectation of the coupling term $\left\langle e^{\tilde{X}^{\mathrm{T}} W X}\right\rangle$ appearing in the action (21, 23–25). The entries W_{ij} must scale as $\mathcal{O}(N^{-1/2})$ to ensure that input fluctuations are independent of N and within the dynamic range of the neurons (see e.g. discussion in 27, section 10.2). Cumulants of W of order 3, as for example present for a log-normal distribution, are hence $\mathcal{O}(N^{-3/2})$ in this scaling. Since mean cross-covariances as well as the variance of covariances are $\mathcal{O}(N^{-1})$ these higher-order cumulants therefore only yield subleading terms, which can be neglected for large network size. This justifies the approximation of any connection matrix W by a Gaussian matrix, given the higher order cumulants are bounded. This is confirmed by the comparison of Erdős-Rényi networks with the Gaussian theory in Fig. 4 in the main text. In Fig. S4 we explicitly test for log-normal weight distributions and show excellent agreement between theory and simulations.

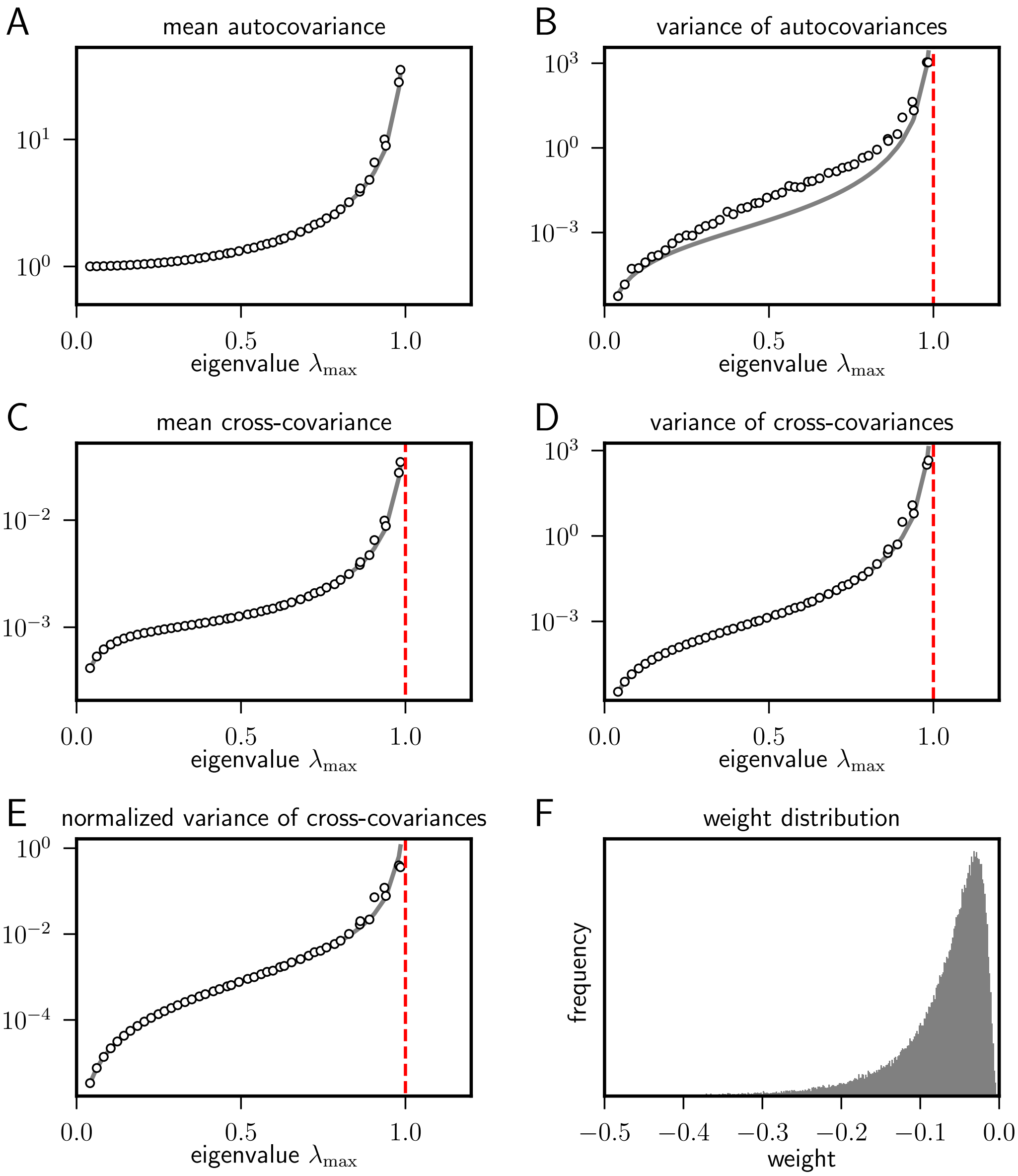

Fig. S4. Log-normal synaptic weight distribution. Mean (absolute value) and variance of auto- (**A**,**B**) and cross-covariances (**C**,**D**), as well as normalized variance of cross-covariances (**E**). Analytical prediction Eqs. (27) and (28) for an inhibitory network with Gaussian connections ($\mu = pw$, $\lambda_{\max}^2 = Np(1-p)w^2$, $w < 0$) and uniform noise autocovariance (solid curve) is compared to numerical evaluation of (12) for inhibitory Erdős-Rényi networks with connection weights that follow a log-normal distribution (circles). Parameters: network size $N = 1000$, connection probability $p = 0.1$, $D = 1$. Mean and standard deviation of connection weights are varied to obtain largest eigenvalues $\lambda_{\max} \in [0, 1]$. (**F**) Log-normal distribution of weights underlying right-most circle in panels A-E.

Effect of heterogeneous firing rates. The theoretical results for the mean and width of the distribution of covariances assume uniform firing rates. These correspond to uniform autocovariances $D_{ii} = D$ of the noisy input in the model of Ornstein-Uhlenbeck processes (10). For these networks, the largest eigenvalue $\lambda_{\max}$ of the effective connectivity W is given by the width of the distribution of spike-count cross-covariances normalized by the mean spike-count autocovariances (Eq. 1 in the main text), which removes the influence of the noise covariance D on cross-covariances. Here, we investigate the influence of distributed firing rates on the estimation of the spectral radius.

Fig. S5F shows the experimentally obtained distribution of spike-count autocovariances. The large width of this distribution, which is on the same order of magnitude as the mean spike-count autocovariance, arises from distributed firing rates (Fig. S5B). This effect is explained by using a geometric series expansion $(\mathcal{I} - W)^{-1} = \mathcal{I} + \sum_{n=1}^{\infty} W^n$ for the expression of integral covariances (12) (13, 30, 31)

$$
\begin{aligned}
c &= (\mathcal{I} - W)^{-1}\, D\, \left(\mathcal{I} - W^{\mathrm{T}}\right)^{-1} \\
&= D + \underbrace{\sum_{n=1}^{\infty} W^n\, D + D \sum_{n=1}^{\infty} W^{\mathrm{T}\,n} + \sum_{m,n=1}^{\infty} W^n\, D\, W^{\mathrm{T}\,m}}_{=:\mathbf{c}^{\mathrm{loc}}} .
\end{aligned} \qquad [29]
$$

Since the noise covariance enters linearly in the equation, mean covariances are unaffected by variability in noise covariances (Fig. S5A,C). Individual autocovariances c_{ii} are primarily determined by D_{ii}. Variability of the latter therefore almost exclusively determines the variance of autocovariances for a large range of $\lambda_{\max}$ (Fig. S5B). Since $D_{ij} = 0$ for $i \neq j$, this leading order effect is absent for cross-covariances. Their variance and the prediction of the spectral radius (Eq. 1 in the main text) are therefore determined by the locally generated covariance c_{ij}^{loc} (see Eq. (29)) and only mildly affected by heterogeneity in firing rates (Fig. S5D,E).

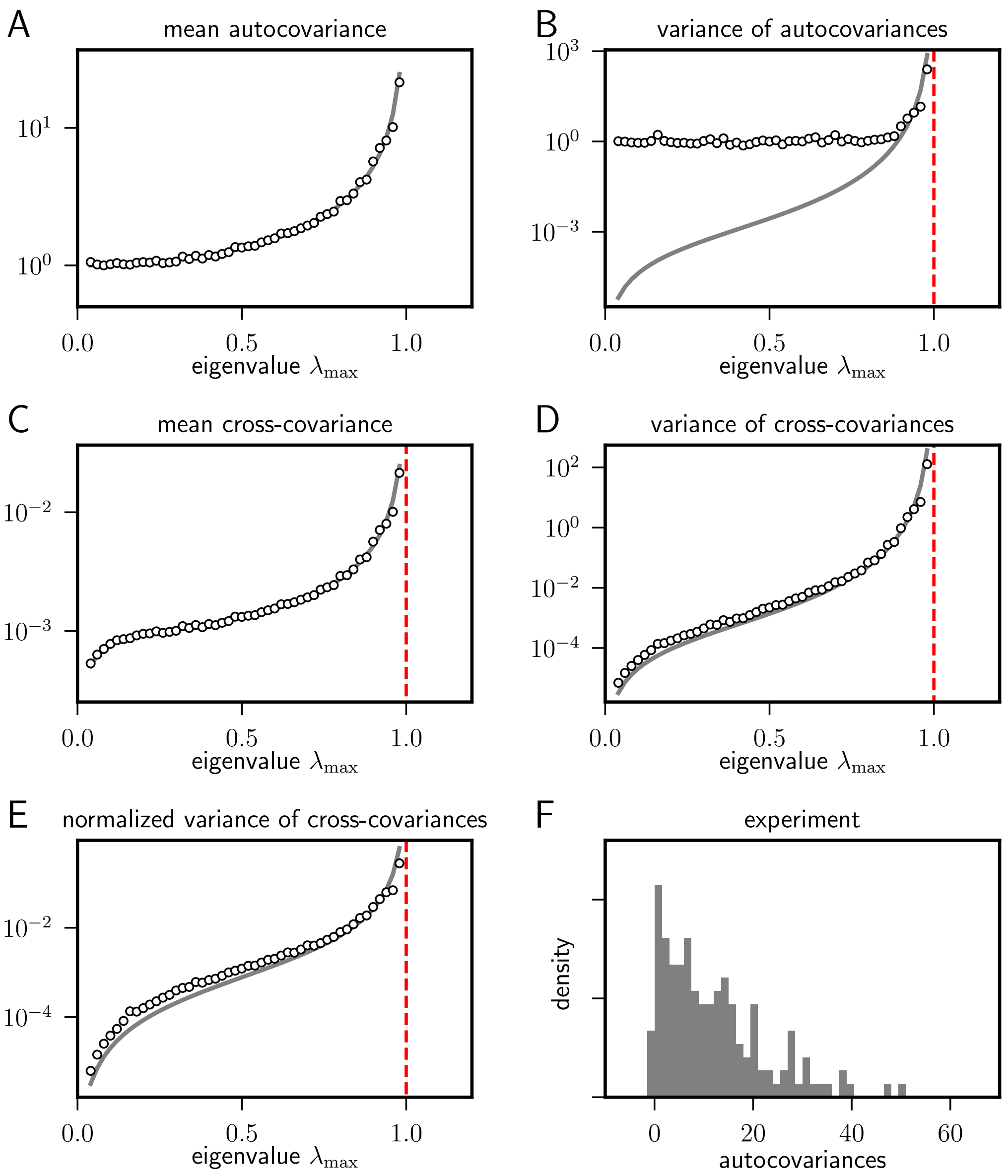

Fig. S5. Heterogeneous firing rates. Mean (absolute value) and variance of auto- (**A**,**B**) and cross-covariances (**C**,**D**), as well as normalized variance of cross-covariances (**E**). Analytical prediction Eqs. (27) and (28) for an inhibitory network with Gaussian connections ($\mu = pw$, $\lambda_{\max}^2 = Np(1-p)w^2$, $w < 0$) and uniform noise autocovariance (solid curve) is compared to numerical evaluation of (12) for inhibitory Erdős-Rényi networks with distributed noise autocovariances (circles). Parameters: network size $N = 1000$, connection probability $p = 0.1$, log-normal distribution of autocovariances with mean and standard deviation $\bar{D} = \delta D = 1$. Weights w of connections are varied to obtain largest eigenvalues $\lambda_{\max} \in [0, 1]$. (**F**) Distribution of spike-count autocovariances $c_{ii} = \frac{1}{T}\left(\langle n_i n_i \rangle - \langle n_i \rangle \langle n_i \rangle\right)$ across trials obtained from experimental data (same dataset as in Fig. 1 of the main text).

Effect of correlated external input. The theory in this study assumes a local network of N neurons that receives uncorrelated input. In cortex, neurons are correlated in part by local connections and in part by overlapping and correlated inter-laminar or inter-area connections from outside the local network (32–34). While the effect of correlated input on mean covariances is investigated in (15), we here also analyze the effect on the distribution of covariances. In case of external input, the dynamics of the local network is given by

$$\frac{dx_i}{dt} = -x_i + \sum_{j=1}^{N} W_{ij} x_j + \sum_{j=1}^{N^{\mathrm{ext}}} M_{ij} y_j + \xi_i,$$

where x are activities of recurrently connected (W) local neurons driven by uncorrelated noise ξ and by correlated input via connections M from an external population of N^{ext} neurons with activities y. The correlated external input adds a non-diagonal contribution to the covariance matrix D

$$c_{ij} = \left[(\mathcal{I} - W)^{-1} \left(D + \underbrace{M c^{\mathrm{ext}} M^{\mathrm{T}}}_{\tilde{D}} \right) \left(\mathcal{I} - W^{\mathrm{T}} \right)^{-1} \right]_{ij} . \qquad [30]$$

Using the geometric series expansion (29) as in (3) with D substituted by $D + \tilde{D} = D + M c^{\mathrm{ext}} M^{\mathrm{T}}$ shows that variability of off-diagonal elements of $M c^{\mathrm{ext}} M^{\mathrm{T}}$ contributes directly to the dispersion of cross-covariances. This effect is not captured by the theory developed in this study. It arises from shared inputs and correlated activity in the external input and is, to leading order, independent of the local connectivity W.

The external input to the local network typically originates from many cortical areas. Therefore, the size of the external population N^{ext} can be assumed to be large and the cross-covariances c_{ij}^{ext} to be small. We here consider the worst case that the external population is of the same size as the local network and exhibits the same potentially strong correlation structure, i.e. $N^{\mathrm{ext}} = N$, $c^{\mathrm{ext}} \sim c$; the effect of $\tilde{D}$ on the covariance structure of the local network is then notable (Fig. S6A-D), especially in the region of small $\lambda_{\max}$. In this region, the local network is only weakly connected and covariances are determined by shared external input. The variability of off-diagonal elements of $\tilde{D}_{ij}$ dominates the locally generated dispersion of cross-covariances c_{ij}^{loc}. The ratio Δ between the variance of cross-covariances and the mean autocovariances, as obtained from the theory derived in this study, does not predict the true ratio of the these quantities (Fig. S6E). However, unlike the distribution of stationary mean activities (see Fig. 3) which is potentially $\mathcal{O}(1)$, the variability of $\tilde{D}_{ij}$ is bounded to be $\mathcal{O}(1/N^{\mathrm{ext}})$. This follows from the $\mathcal{O}(1/\sqrt{N^{\mathrm{ext}}})$ scaling of the mean and standard deviation of the external synapses M_{ij}, which ensures that input fluctuations are independent of N^{ext} and within the dynamic range of the neurons in the local network (27, section 10.2). Therefore, for larger $\lambda_{\max}$, locally generated covariances c_{ij}^{loc} dominate the direct influence $\tilde{D}_{ij}$ of external correlations. Even though the mean and dispersion of covariances remain affected by the external input, their effect approximately cancels in the ratio Δ. As a result, in this regime, the relation between $\lambda_{\max}$ of the local network and the ratio Δ is again accurately predicted by the current theory (Fig. S6E). For a more realistic, larger external population or less correlated external input, the deviation from the theory becomes even smaller. Therefore, a large ratio Δ, as observed in the experimental data, can only be caused by a large $\lambda_{\max}$ of the local connectivity. This shows that our main conclusion is robust against the presence of correlated inputs under sensible assumptions.

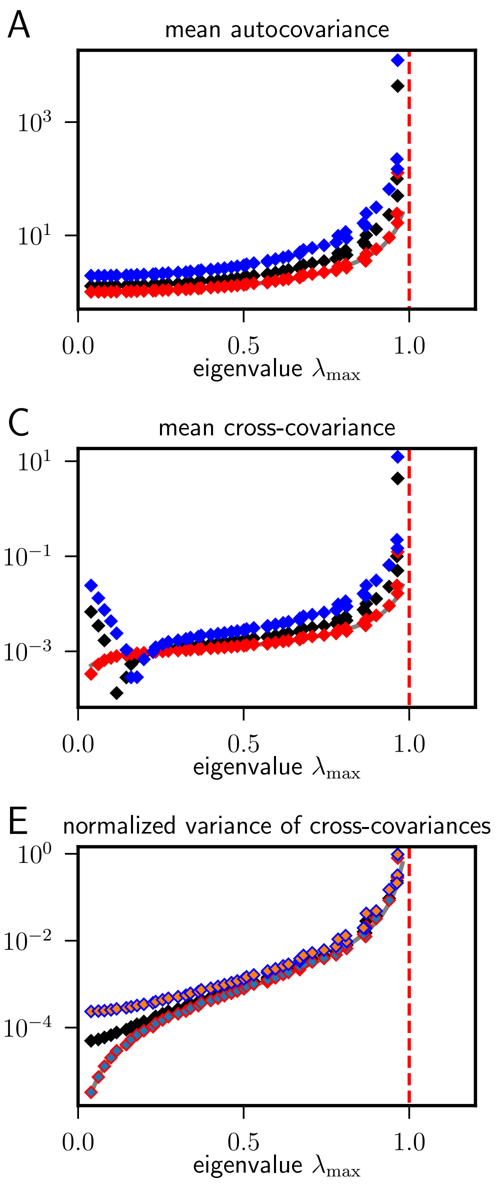

Fig. S6. Correlated external input. Mean (absolute value) and variance of auto- (**A**,**B**) and cross-covariances (**C**,**D**), as well as normalized variance of cross-covariances (**E**). Analytical prediction (27) and (28) for an inhibitory network with Gaussian connectivity ($\mu = pw$, $\lambda_{\max}^2 = Np(1-p)w^2$ with size N, largest eigenvalue $\lambda_{\max}$) and uniform noise autocovariance D (solid curve) is compared to numerical evaluation of (12) for inhibitory Erdős-Rényi networks (symbols) with uniform stationary mean activity D, receiving correlated input from an external population ($N^{\text{ext}} = N$). Red, black, and blue symbols show weak ($\lambda_{\max}^M = 0.08$), intermediate ($\lambda_{\max}^M = 0.52$) and strong ($\lambda_{\max}^M = 0.96$) connectivity M between populations, respectively. Note that the mean cross-covariance changes sign in the low-$\lambda_{\max}$ regime for sufficiently strong external input. Parameters: network size $N = 1000$, connection probability $p = 0.1$, $D = 1$. Weights w of connections are varied to obtain largest eigenvalues $\lambda_{\max} \in [0, 1]$.

Effect of correlated connections. The theory in this study assumes independent connections. There is, however, experimental evidence for non-random features of network connectivity (see e.g. (35–37)). Due to the $\mathcal{O}(1/\sqrt{N})$ scaling of connection weights ((27, section 10.2), see section 3), only connection statistics involving one or two connections are relevant as the influence of higher order motifs is suppressed by the large network size. The dominant departure from random connectivity are reciprocal connections of which a surplus was observed experimentally (36). Including these in the cumulant expansion yields

$$\left\langle e^{\tilde{X}^{\mathrm{T}} W X} \right\rangle = e^{\mu \sum_{i,j=1}^{N} \tilde{X}_i X_j + \frac{\sigma^2}{N} \sum_{i,j=1}^{N} \tilde{X}_i \tilde{X}_i X_j X_j} \cdot e^{\frac{\lambda\sigma^2}{N} \sum_{i,j=1}^{N} \tilde{X}_i X_i \tilde{X}_j X_j},$$

where $\lambda\sigma^2/N$ denotes the second cumulant of reciprocal connections. This term qualitatively yields new effects as it leads to a renormalization of the response function, known from spin glasses (20), recently investigated in the context of neuronal networks (38). Furthermore, symmetry in the network connectivity affects the spectral radius of the connectivity matrix (39), which is a key component in our prediction of the operational regime. In the following, we test sensitivity of our result to inclusion of reciprocal connections.

Let us assume the joint probability of two connections is given by Fig. S7F, where $\rho \in [0, 1]$ determines the degree of symmetry of connections: For $\rho = p$, we obtain the special case of independent connections, which is considered in the main text. $\rho > p$ indicates a surplus of reciprocal connections with $\rho = 1$ being a fully symmetric network, $\rho = 0$ is the limit of no reciprocal connections. The parameter ρ can be estimated from the experimental data of (36) as the ratio between the number of observed reciprocal connections $N_{\leftrightarrow}$ and the total number of observed connections N_{tot} divided by p

$$\rho = \frac{1}{p} \frac{N_{\leftrightarrow}}{N_{\mathrm{tot}}} = \frac{1}{0.116} \cdot \frac{218}{3312 + 495 + 218} \approx 0.47 > p, \qquad [31]$$

showing that reciprocal connections are over-represented. The variance of connections is by construction

$$\sigma^2/N = w^2 \left(\langle W_{ij} W_{ij} \rangle - \langle W_{ij} \rangle^2 \right) = p(1-p)w^2$$

independent of ρ. The covariance of connections is given by $\lambda\sigma^2/N = (\langle W_{ij} W_{ji} \rangle - \langle W_{ij} \rangle \langle W_{ji} \rangle)\, w^2 = p\,(\rho - p)\, w^2$ resulting in $\lambda = \frac{\rho - p}{1-p} \approx 0.4$ for the ratio between the covariance of reciprocal connections and the variance of connections (39). Numerical simulations of networks with the statistics of reciprocal connections as in Fig. S7F with ρ given by Eq. (31) show that the first two cumulants of the covariance distributions are still in qualitative agreement with those in a completely random network (Fig. S7). Even for completely symmetric networks, deviations from the theory of random networks are not affecting the scale of these measures. The model prediction for λ_{max} derived from the experimental data close to 1 is therefore robust against such correlations in connections.

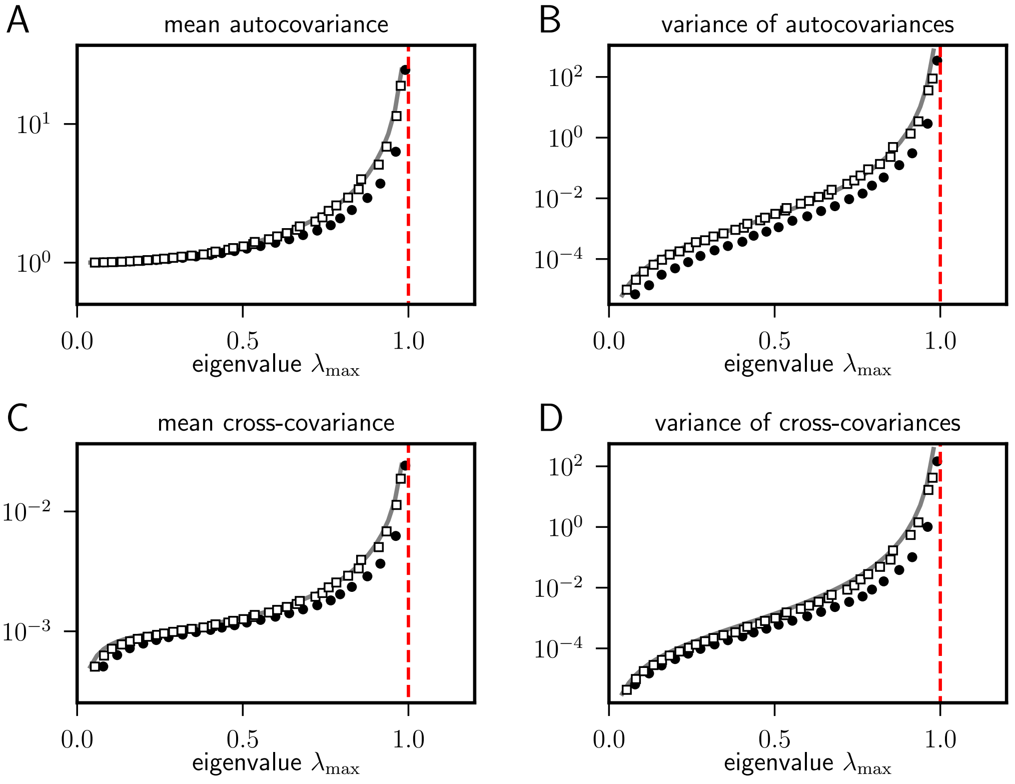

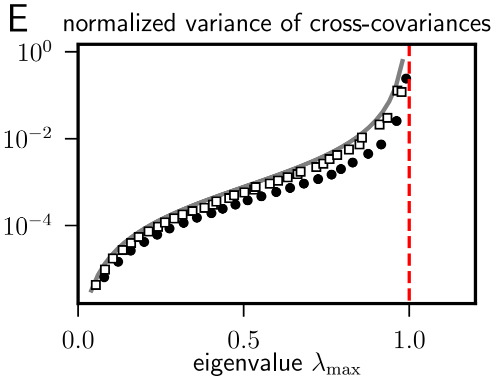

F

$\leftarrow \backslash \rightarrow$	0	1
0	$1-(2-\rho)p$	$(1-\rho)p$
1	$(1-\rho)p$	ρp

Fig. S7. Correlated connections. Mean (absolute value) and variance of auto- (**A**,**B**) and cross-covariances (**C**,**D**), as well as normalized variance of cross-covariances (**E**). Analytical prediction (27) and (28) for an inhibitory network with Gaussian connectivity ($\mu = pw$, $\lambda_{\max}^2 = Np(1-p)w^2$ and $\rho = p$, see panel (**F**), corresponding to completely randomly drawn connections) with uniform noise autocovariance D (solid curve) is compared to numerical evaluation of (12) for inhibitory Erdős-Rényi networks (symbols) with joint statistics of reciprocal connections (**F**) with $\rho = 0.47$ (extracted from (36); squares) or $\rho = 1$ (fully symmetric; dots). Arrows in (**F**) denote the direction of the connection between two neurons, 0 and 1 the presence or absence of connections with probabilities given in the table. Parameters: network size $N = 1000$, connection probability $p = 0.1$, $D = 1$. Weights w of connections are varied to obtain largest eigenvalues $\lambda_{\max} \in [0, 1]$.

4. Numerical simulations

Figure 2. To demonstrate multi-neuron coordinated activity in different state space projections, we consider a simple network model with a sparsely and randomly connected population of size $N = 1000$ and covariance of single-unit fluctuations $D = 1$. Connections $W_{ij} \overset{\text{i.i.d.}}{\sim} \mathcal{B}(p, w)$ are drawn independently from a Bernoulli distribution with connection probability $p = 0.1$ and weight $w = 9.99/N$ (panels A and B) or $w = -3.1/\sqrt{N}$ (panels C and D).

Figure 3. To illustrate the general mechanism relating network heterogeneity, eigenvalues, and distributions of covariances, we consider a simple network model with a sparsely and randomly connected inhibitory population of size $N = 1000$ and covariance of single-unit fluctuations $D = 1$. Connections $W_{ij} \overset{\text{i.i.d.}}{\sim} \mathcal{B}(p, w)$ are drawn independently from the same Bernoulli distribution with connection probability $p = 0.1$ and weight $w = -1/\sqrt{N}$ (first row), $w = -2/\sqrt{N}$ (second row), $w = -2.5/\sqrt{N}$ (third row) and $w = -3/\sqrt{N}$ (fourth row).

Figure 4. All networks have sparse random connections of uniform strength drawn from Bernoulli distributions. Furthermore, neurons have a fixed number of incoming connections (in-degree) and do neither connect to themselves (no autapses) nor are they connected multiple times to other neurons (no multapses). The maximum eigenvalue on the abscissa (panels A-C) is varied by the choice of the connection strength.

Homogeneous inhibitory network: We consider inhibitory populations of size $N = 1000$ (panels A,B) or $N = 10000$ (panels C,E), $D = 1$, connection probability $p = 0.1$ and uniform non-zero weights varied in the range $w = -0.1, ..., -0.001$ (panels A,B) or $w = -0.03, ..., -0.003$ (panel C). Panel E shows data for $w = -0.0285$.

Excitatory-inhibitory network: We consider a network of $N_E = 8000$ excitatory and $N_I = 2000$ inhibitory neurons with $D = 1$, connection probability $p = 0.1$, uniform excitatory connection strengths varied in the range $w_E = 0.001, ..., 0.01$ and uniform inhibitory connection strengths varied in the range $w_I = -0.06, ..., -0.006$. Panel F shows data for $w_E = 0.009$ and $w_I = -0.05$.

Inhibitory network with distance-dependent connectivity: We consider a network of $N = 10000$ inhibitory neurons ($D = 1$) randomly positioned on a 1 mm × 1 mm sheet. Each neuron receives $K = 100$ incoming connections of uniform strength varied in the range $w = -0.1, ..., -0.01$ depending on the desired spectral radius λ_{max}. Connections are drawn from a connection profile $p(x) \sim \exp(-x^2/(2\sigma_{\text{conn}}^2))$ where x is the Euclidean distance between the presynaptic and postsynaptic neuron, and $\sigma_{\text{conn}} = 50\,\mu\text{m}$ the space constant.

Leaky integrate-and-fire network: We consider a network of $N = 10000$ inhibitory leaky integrate-and-fire (LIF) model neurons with delta-shaped postsynaptic currents. The membrane potential of each neuron follows the differential equation

$$\tau_m \frac{dV_i}{dt} = -V_i + \tau_m \sum_j J_{ij} s_j (t - h) ,$$

where $s_j(t) = \sum_k \delta\left(t - t_k^j\right)$ is the spike train of the j-th neuron and t_k^j denotes the k-th spike of neuron j, which occurs whenever V_j exceeds the threshold θ. The membrane potential is reset $V_j(t_k^j+) \leftarrow V_r$ to the reset potential V_r after each such event and held at this level for the absolute refractory time τ_r. The time lag h equals the resolution of the time-driven simulation. We choose a connection probability $p = 0.1$ and uniform weights $J \in [-1.1\,\text{mV}, -0.1\,\text{mV}]$. Neuron and simulation parameters are shown in Tab. S1.

Figure 6. Linear instability is determined by an eigenvalue close to the critical line $\text{Re}(\lambda) = 1$. For the dynamically balanced network close to the critical point (Fig. 4 left) we consider a sparse, random network of $N = 5000$ inhibitory neurons with independent and identically distributed connections $W_{ij} \overset{\text{i.i.d.}}{\sim} \mathcal{B}(p, w)$. This network by definition has an inhibition-dominated feedback. The connection probability $p = 0.1$ and weight $w = -3.05/\sqrt{N}$ are chosen such that the theoretical prediction for the largest bulk eigenvalue is $\lambda_{\text{max}} = \sqrt{Np(1-p)w^2} = 0.915 \lessapprox 1$.

For networks with almost vanishing feedback (Fig. 4 right), we consider a sparse, random network of $N = 5000$ excitatory neurons with a feedback of order $1 \ll N$, where N is the network size. Connections $W_{ij} \overset{\text{i.i.d.}}{\sim} \mathcal{B}(p, w)$ are independent and identically distributed. The connection probability $p = 0.1$ and weight $w = 9.95/N$ are chosen such that the feedback $N\mu = Npw = 0.995 \lessapprox 1$ almost compensates the neuronal leak (Eq. (7)). The critical eigenvalue in this network is given by the feedback $N\mu$ and corresponds to the population activity.

5. Code availability

The code for the numerical simulations is available from the authors upon request.

6. Data availability

The dataset analyzed in the current study is published fully annotated and with loading and analysis software in a scientific data publication (40).

Table S1. Specification of neuron and simulation parameters for network of LIF model neurons shown in Fig. 4 of the main text.

**Neuron model**		
**Name**	**Value**	**Description**
τ_{m}	20 ms	membrane time constant
τ_{r}	2 ms	absolute refractory period
V_{r}	0 mV	reset potential
θ	15 mV	fixed firing threshold
E_{leak}	0 mV	leak potential
**Simulation parameters**		
**Name**	**Value**	**Description**
h	0.1 ms	simulation time step
T	1000s	simulation time after initial transients
T_{trial}	1s	time window to compute spike counts

References

1. Barlow R (2013) *Statistics: A Guide to the Use of Statistical Methods in the Physical Sciences.* (John Wiley & Sons).
2. Lehmann EL, Casella G (2006) *Theory of point estimation.* (Springer Science & Business Media).
3. Ecker AS, Berens P, Keliris GA, Bethge M, Logothetis NK (2010) Decorrelated neuronal firing in cortical microcircuits. *Science* 327(5965):584–587.
4. Sompolinsky H, Crisanti A, Sommers HJ (1988) Chaos in random neural networks. *Phys. Rev. Lett.* 61(3):259–262.
5. van Vreeswijk C, Sompolinsky H (1996) Chaos in neuronal networks with balanced excitatory and inhibitory activity. *Science* 274:1724–1726.
6. Aljadeff J, Stern M, Sharpee T (2015) Transition to chaos in random networks with cell-type-specific connectivity. *Phys. Rev. Lett.* 114(8):088101.
7. Kadmon J, Sompolinsky H (2015) Transition to chaos in random neuronal networks. *Phys. Rev. X* 5(4):041030.
8. Schuecker J, Goedeke S, Helias M (2018) Optimal sequence memory in driven random networks. *Physical Review X* 8(4):041029.
9. Crisanti A, Sompolinksy H (2018) Path integral approach to random neural networks. *Phys. Rev. E* 98(6):062120.
10. Grytskyy D, Tetzlaff T, Diesmann M, Helias M (2013) A unified view on weakly correlated recurrent networks. *Front. Comput. Neurosci.* 7:131.
11. Ginzburg I, Sompolinsky H (1994) Theory of correlations in stochastic neural networks. *Phys. Rev. E* 50(4):3171–3191.
12. Renart A, et al. (2010) The asynchronous state in cortical circuits. *Science* 327:587–590.
13. Trousdale J, Hu Y, Shea-Brown E, Josic K (2012) Impact of network structure and cellular response on spike time correlations. *PLOS Comput. Biol.* 8(3):e1002408.
14. Tetzlaff T, Helias M, Einevoll GT, Diesmann M (2012) Decorrelation of neural-network activity by inhibitory feedback. *PLOS Comput. Biol.* 8(8):e1002596.
15. Helias M, Tetzlaff T, Diesmann M (2014) The correlation structure of local cortical networks intrinsically results from recurrent dynamics. *PLOS Comput. Biol.* 10(1):e1003428.
16. De Dominicis C (1978) Dynamics as a substitute for replicas in systems with quenched random impurities. *Phys. Rev. B* 18(9):4913.
17. Chow C, Buice M (2015) Path integral methods for stochastic differential equations. *The Journal of Mathematical Neuroscience* 5(8).
18. Hertz JA, Roudi Y, Sollich P (2017) Path integral methods for the dynamics of stochastic and disordered systems. *Journal of Physics A: Mathematical and Theoretical* 50(3):033001.
19. Moshe M, Zinn-Justin J (2003) Quantum field theory in the large n limit: a review. *Physics Reports* 385:69–228.
20. Sompolinsky H, Zippelius A (1982) Relaxational dynamics of the edwards-anderson model and the mean-field theory of spin-glasses. *Phys. Rev. B* 25(11):6860–6875.
21. Fischer KH, Hertz JA (1991) *Spin Glasses.* (Cambridge University Press).
22. Gardiner CW (1985) *Handbook of Stochastic Methods for Physics, Chemistry and the Natural Sciences*, Springer Series in Synergetics. (Springer-Verlag, Berlin) No. 13, 2nd edition.
23. Sherrington D, Kirkpatrick S (1975) Solvable model of a spin-glass. *Phys. Rev. Lett.* 35:1792–1796.
24. De Dominicis C, Peliti L (1978) Field-theory renormalixation and critical dynamics above t_c: Helium, antiferromagnets, and liquid-gas systems. *Phys. Rev. B* 18(1):353–376.
25. Nishimori H (2001) *Statistical Physics of Spin Glasses and Information Processing An Introduction.* (Clarendron Press, Oxford).
26. Gardiner CW (1983) *Handbook of Stochastic Methods for Physics, Chemistry and the Natural Sciences*, Springer Series in Synergetics. (Springer-Verlag, Berlin) No. 13.
27. van Vreeswijk C, Sompolinsky H (1998) Chaotic balanced state in a model of cortical circuits. *Neural Comput.* 10:1321–1371.
28. Amit DJ (2005) *Field Theory, the Renormalization Group, and Critical Phenomena; 3rd ed.* (World Scientific, Singapore).
29. Roudi Y, Hertz J (2011) Mean field theory for nonequilibrium network reconstruction. *Physical Review Letters* 106(4):048702.
30. Pernice V, Staude B, Cardanobile S, Rotter S (2011) How structure determines correlations in neuronal networks. *PLOS Comput. Biol.* 7(5):e1002059.
31. Pernice V, Staude B, Cardanobile S, Rotter S (2012) Recurrent interactions in spiking networks with arbitrary topology. *Phys. Rev. E* 85(3):031916.
32. Abeles M (1982) *Local Cortical Circuits: An Electrophysiological Study*, Studies of Brain Function. (Springer-Verlag, Berlin, Heidelberg, New York).
33. Binzegger T, Douglas RJ, Martin KAC (2004) A quantitative map of the circuit of cat primary visual cortex. *J. Neurosci.* 39(24):8441–8453.
34. Stepanyants A, Martinez LM, Ferecskó AS, Kisvárday ZF (2009) The fractions of short- and long-range connections in the visual cortex. *Proc. Natl. Acad. Sci. USA* 106(9):3555–3560.
35. Sporns O, Kötter R (2004) Motifs in brain networks. *PLoS Biol* 2(11):e369.
36. Song S, Sjöström P, Reigl M, Nelson S, Chklovskii D (2005) Highly nonrandom features of synaptic connectivity in local cortical circuits. *PLOS Biol.* 3(3):e68.
37. Reimann MW, Horlemann AL, Ramaswamy S, Muller EB, Markram H (2017) Morphological diversity strongly constrains synaptic connectivity and plasticity. *Cereb. Cortex* 27(9):4570–4585.

38. Martí D, Brunel N, Ostojic S (2018) Correlations between synapses in pairs of neurons slow down dynamics in randomly connected neural networks. *Phys. Rev. E* 97(6):062314.
39. Sommers H, Crisanti A, Sompolinsky H, Stein Y (1988) Spectrum of large random asymmetric matrices. *Phys. Rev. Lett.* 60(19):1895–1898.
40. Brochier T, et al. (2018) Massively parallel recordings in macaque motor cortex during an instructed delayed reach-to-grasp task. *Scientific Data* 5:180055.
